# Assisted Reproductive Technology Surveillance — United States, 2014

**DOI:** 10.15585/mmwr.ss6606a1

**Published:** 2017-02-10

**Authors:** Saswati Sunderam, Dmitry M. Kissin, Sara B. Crawford, Suzanne G. Folger, Denise J. Jamieson, Lee Warner, Wanda D. Barfield

**Affiliations:** 1 Division of Reproductive Health, National Center for Chronic Disease Prevention and Health Promotion, CDC

## Abstract

**Problem/Condition:**

Since the first U.S. infant conceived with assisted reproductive technology (ART) was born in 1981, both the use of ART and the number of fertility clinics providing ART services have increased steadily in the United States. ART includes fertility treatments in which eggs or embryos are handled in the laboratory (i.e., in vitro fertilization [IVF] and related procedures). Women who undergo ART procedures are more likely than women who conceive naturally to deliver multiple-birth infants. Multiple births pose substantial risks to both mothers and infants, including obstetric complications, preterm delivery, and low birthweight infants. This report provides state-specific information for the United States (including the District of Columbia and Puerto Rico) on ART procedures performed in 2014 and compares birth outcomes that occurred in 2014 (resulting from ART procedures performed in 2013 and 2014) with outcomes for all infants born in the United States in 2014.

**Period Covered:**

2014.

**Description of System:**

In 1996, CDC began collecting data on ART procedures performed in fertility clinics in the United States as mandated by the Fertility Clinic Success Rate and Certification Act of 1992 (FCSRCA) (Public Law 102–493). Data are collected through the National ART Surveillance System (NASS), a web-based data collection system developed by CDC. This report includes data from 52 reporting areas (the 50 states, the District of Columbia, and Puerto Rico).

**Results:**

In 2014, a total of 169,568 ART procedures (range: 124 in Wyoming to 21,018 in California) with the intent to transfer at least one embryo were performed in 458 U.S. fertility clinics and reported to CDC. These procedures resulted in 56,028 live-birth deliveries (range: 52 in Wyoming to 7,230 in California) and 68,782 infants born (range: 64 in Wyoming to 8,793 in California). Nationally, the total number of ART procedures performed per million women of reproductive age (15–44 years), a proxy measure of the ART usage rate, was 2,647 (range: 364 in Puerto Rico to 6,726 in Massachusetts). ART use exceeded the national average in 13 reporting areas (Connecticut, Delaware, the District of Columbia, Hawaii, Illinois, Maryland, Massachusetts, New Hampshire, New Jersey, New York, Pennsylvania, Rhode Island, and Virginia). Eight reporting areas (Connecticut, the District of Columbia, Hawaii, Illinois, Maryland, Massachusetts, New Jersey, and New York) had rates of ART use exceeding 1.5 times the national average.

Nationally, among ART transfer procedures in patients using fresh embryos from their own eggs, the average number of embryos transferred increased with increasing age of the woman (1.7 among women aged <35 years, 1.9 among women aged 35–37 years, and 2.3 among women aged >37 years). Among women aged <35 years, who typically are considered to be good candidates for elective single embryo transfer (eSET) procedures, the national eSET rate was 28.5% (range: 4.3% in Puerto Rico to 67.9% in Delaware).

In 2014, ART contributed to 1.6% of all infants born in the United States (range: 0.4% in Puerto Rico to 4.7% in Massachusetts) and 18.3% of all multiple-birth infants (range: 5.5% in Alaska and West Virginia to 37.3% in Hawaii), including 18.0% of all twin infants (range: 5.2% in some states to 36.2% in Hawaii) and 26.4% of all triplets and higher-order infants (range: 0% in some states to 65.2% in Hawaii). Percentages of live births that were multiple-birth deliveries were higher among infants conceived with ART (39.4%; range: 11.5% in Delaware to 55.6% in Puerto Rico) than among all infants born in the total birth population (3.5%; range: 2.2% in Puerto Rico to 4.4% in New Jersey). Approximately 38.0% of ART-conceived infants were twin infants, and 2.0% were triplets and higher-order infants. ART-conceived twins accounted for approximately 95.3% of all ART-conceived infants born in multiple deliveries.

Nationally, infants conceived with ART contributed to 5.5% of all low birthweight (<2,500 g) infants (range: 1.2% in West Virginia to 14.2% in Massachusetts). Among ART-conceived infants, 27.8% were low birthweight (range: 10.6% in Delaware to 44.4% in Puerto Rico), compared with 8.0% among all infants (range: 5.9% in Alaska to 11.3% in Mississippi).

ART-conceived infants contributed to 4.7% of all preterm (<37 weeks) infants (range: 1.2% in Puerto Rico to 13.4% in Massachusetts). Percentages of preterm births were higher among infants conceived with ART (33.2%; range: 18.9% in the District of Columbia to 45.9% in Puerto Rico) than among all infants born in the total birth population (11.3%; range: 8.5% in California to 16.0% in Mississippi).

The percentage of ART-conceived infants who were low birthweight was 8.9% (range: 3.2% in some states to 16.1% in Vermont) among singletons and 55.2% (range: 38.5% in Delaware to 77.8% in Alaska) among twins; the corresponding percentages of low birthweight infants among all infants born were 6.3% for singletons (range: 4.6% in Alaska, North Dakota, and Oregon to 9.5% in Puerto Rico) and 55.2% for twins (range: 46.1% in Alaska to 65.6% in Mississippi).

The percentage of ART-conceived infants who were preterm was 13.2% (range: 7.5% in Rhode Island to 23.4% in West Virginia) among singletons and 62.2% (range: 33.3% in some states to 81.4% in Mississippi) among twins; the corresponding percentages of preterm infants among all infants were 9.7% for singletons (range: 1.7% in the District of Columbia to 14.2% in Mississippi) and 56.6% for twins (range: 47.2% in Vermont to 66.9% in Wyoming).

**Interpretation:**

The percentage of infants conceived with ART varied considerably by reporting area. Multiple births from ART contributed to a substantial proportion of all twins, triplets, and higher-order infants born. Low birthweight and preterm infant birth rates were disproportionately higher among ART-conceived infants than among the overall birth population. Although women aged <35 years are typically considered good candidates for eSET, on average two embryos were transferred per ART procedure with women in this group. Compared with ART-conceived singletons, ART-conceived twins were approximately five times more likely to be born preterm and approximately six times more likely to be born with low birthweight. Singleton infants conceived with ART had higher percentages of preterm birth and low birthweight than all singleton infants born in the United States. ART use per population unit was geographically variable, with 13 reporting areas showing ART use higher than the national rate. Of the four states (Illinois, Massachusetts, New Jersey, and Rhode Island) with comprehensive statewide-mandated health insurance coverage for ART procedures (i.e., coverage for at least four cycles of IVF), three (Illinois, Massachusetts, and New Jersey) had rates of ART use exceeding 1.5 times the national rate. This type of mandated insurance has been associated with greater use of ART and likely accounts for some of the difference in per capita ART use observed among states.

**Public Health Action:**

Reducing the number of embryos transferred and increasing use of eSET when clinically appropriate could help reduce multiple births and related adverse health consequences. Because twins account for the majority of ART-conceived multiple births, improved provider practices and patient education and counseling on the maternal and infant health risks of having twins are needed. Although ART contributes to high percentages of multiple births, other factors not investigated in this report (e.g., delayed childbearing and use of non-ART fertility treatments) also contribute to multiple births and warrant further study.

## Introduction

Since the birth of the first U.S. infant conceived with assisted reproductive technology (ART) in 1981, use of advanced technologies to overcome infertility has increased, as has the number of fertility clinics providing ART services and procedures in the United States (*1*). In 1992, Congress passed the Fertility Clinic Success Rate and Certification Act (FCSRCA) (Public Law 102–493), which requires that all U.S. fertility clinics performing ART procedures report data to CDC annually on every ART procedure performed. CDC initiated data collection in 1996 and published the first annual ART Success Rates Report in 1997 (*2*). Several measures of success for ART are presented in the annual report (*1*,*3*), including the percentage of ART procedures and transfers that result in pregnancies, live-birth deliveries, singleton live-birth deliveries, and multiple live-birth deliveries.

Although ART helps millions of infertile couples to achieve pregnancy, it is associated with potential health risks to both mother and infant. Because multiple embryos are transferred in the majority of ART procedures, ART often results in multiple-gestation pregnancies and multiple births (*4*–*11*). Risks to the mother from multiple births include higher rates of caesarean deliveries, maternal hemorrhage, pregnancy-related hypertension, and gestational diabetes (*12*,*13*). Risks to the infant include prematurity, low birthweight, infant death, and elevated risk for birth defects and developmental disability (*4*–*18*). Further, even singleton infants conceived with ART have a higher risk for low birthweight and prematurity than singletons not conceived with ART (*19*,*20*).

This report was compiled on the basis of ART surveillance data reported to CDC’s Division of Reproductive Health for procedures performed in 2014. Data on the use of ART are presented for residents of each U.S. state, the District of Columbia, and Puerto Rico; data also are reported for outcomes for infants born in 2014 resulting from ART procedures performed in 2013 and 2014. The report also examines the contribution of ART to select outcomes (i.e., multiple-birth infants, low birthweight infants, and preterm infants) and compares ART infant outcomes with outcomes among all infants born in the United States (including Puerto Rico) in 2014.

## Methods

### National ART Surveillance System

In 1996, CDC initiated data collection of ART procedures performed in the United States. ART data for 1995–2003 were obtained from the Society for Assisted Reproductive Technology (SART). Since 2004, CDC has contracted with Westat, Inc., a statistical survey research organization, to obtain data from all fertility clinics in the United States through the National ART Surveillance System (NASS), a web-based data collection system developed by CDC (https://www.cdc.gov/art/nass/index.html). Clinics can enter their data directly into NASS and verify its accuracy before sending the data to Westat. SART-member clinics can report their data to NASS through SART. Non-SART-member clinics can report their data directly through NASS. The data then are compiled by Westat and reviewed by both CDC and Westat. A small proportion of clinics (7.8%) did not report their data to CDC in 2014 and are listed as nonreporting programs in the 2014 ART Fertility Clinic Success Rates Report, as required by FCSRCA. Because nonreporting clinics tend to be smaller on average than reporting clinics, NASS is estimated to contain information on 98.0% of all ART procedures in the United States (*1*).

Data collected include patient demographics, medical history, and infertility diagnoses; clinical information pertaining to the ART procedure type; and information regarding resultant pregnancies and births. The data file contains one record per ART procedure (or cycle of treatment) performed. Because ART providers typically do not provide continued prenatal care after a pregnancy is established, information on live births for all procedures is collected by ART clinics either directly from the patients (73.7%) or from the patients’ obstetric providers (25.2%). Approximately 1.0% of pregnancy outcomes are missing in NASS.

### ART Procedures

ART includes fertility treatments in which eggs or embryos are handled in a laboratory (e.g., in vitro fertilization [IVF], gamete intrafallopian transfer, and zygote intrafallopian transfer). Approximately 99.0% of ART procedures performed are IVF. Because an ART procedure consists of several steps over an interval of approximately 2 weeks, a procedure often is referred to as a cycle of treatment. An ART cycle usually begins with drug-induced ovarian stimulation. If eggs are produced, the cycle progresses to the egg-retrieval stage, which involves surgical removal of the eggs from the ovaries. After the eggs are retrieved, they are combined with sperm in the laboratory during the IVF procedure. If successful, the most viable embryos (i.e., those that appear morphologically most likely to develop and implant) are selected for transfer back into the uterus by clinicians. If an embryo implants in the uterus, a clinical pregnancy is diagnosed by the presence of a gestational sac detectable by ultrasound. The majority of pregnancy losses occur within the first 12 weeks. Beyond 12 weeks of gestation, the pregnancy usually progresses to a live-birth delivery, defined as the delivery of one or more live-born infants. Survival of pregnancy ranges from 95.0% at 16 weeks to 98.0% at 20 weeks (*21*). ART does not include treatments in which only sperm are handled (i.e., intrauterine insemination) or procedures in which a woman takes drugs to stimulate egg production without the intention of having eggs retrieved.

ART procedures are classified into four types on the basis of the source of the egg (patient or donor) and the status of the embryos (fresh or thawed). Both fresh and thawed embryos can be derived from either the patient’s eggs or the donor’s eggs. Both patient and donor embryos can be created using sperm from a partner or from a donor. ART procedures involving fresh embryos include an egg-retrieval stage. ART procedures that use thawed embryos do not include egg retrieval because the eggs were fertilized during a previous procedure and the resultant embryos were frozen until the current procedure. An ART cycle can be discontinued at any step for medical reasons or by patient choice.

### Variables and Definitions

ART data and outcomes from ART procedures are presented by patient’s residence (i.e., reporting area) at the time of treatment, which might not be the same as the location where the procedure was performed. If information on patient’s residence was missing, residence was assigned as the location where the procedure was performed. ART procedures performed in the United States among non-U.S. residents are included in NASS data; however, they were excluded from certain calculations for which the exact denominators were not known. To protect confidentiality in the presentation of data in tables, cells with values of 1–4 are suppressed, as are data that can be used to derive cell values of 1–4; however, these values are included in the totals. In some cases as applicable, states are not identified when reporting ranges to protect confidentiality. Because of small numbers, ART data from U.S. territories (with the exception of Puerto Rico) are not included in this report. In addition, estimates derived from cell values <20 in the denominator have been suppressed because they are unstable, and estimates could not be calculated when the denominator was zero (e.g., preterm birth among triplets, in reporting areas with no triplet births).

This report presents data on all procedures initiated with the intent to transfer at least one embryo; however, birth outcomes are determined on the basis of procedures that involved embryo transfer. The number of ART procedures performed per 1 million women of reproductive age (15–44 years) was calculated and the resulting rate approximates the proportion of women of reproductive age who used ART in each reporting area. However, this proxy measure of ART use is only an approximation because some women who used ART might fall outside the age range of 15–44 years (approximately 10.0% in 2014) and some women might have had more than one procedure during the reporting period.

A live-birth delivery was defined as birth of one or more live-born infants. A singleton live-birth delivery was defined as a birth of only one infant who was born live. A multiple live-birth delivery was defined as a birth of two or more infants, at least one of whom was born live. Low birthweight was defined as <2,500 g and very low birthweight as <1,500 g. For comparability with births to women who did not undergo ART, for whom gestational age is determined on the basis of the date of the last menstrual period, gestational age for fresh ART procedures was calculated by subtracting the date of egg retrieval from the birth date and adding 14 days. For frozen embryo cycles and for fresh ART procedures for which the date of retrieval was not available, gestational age was calculated by subtracting the date of embryo transfer from the birth date and adding 17 days (to account for an average of 3 days in embryo culture). Preterm delivery was defined as gestational age <37 weeks and very preterm delivery as gestational age <32 weeks (*22*).

Elective single-embryo transfer (eSET) is a procedure in which one embryo, selected from a larger number of available embryos, is placed in the uterus, with extra embryos cryopreserved. Fresh transfer procedures in which only one embryo was transferred but no embryos were cryopreserved are considered single-embryo transfer (SET) but not considered eSET. In this report, percentages of eSET procedures and the average number of embryos transferred were calculated for patients who used fresh embryos from their own eggs, in which at least one embryo was transferred. The rate of eSET was calculated by dividing the total number of transfer procedures, in which only one embryo was transferred and one or more embryos were cryopreserved, by the sum of the total number of single-embryo transfer procedures where extra embryos were cryopreserved plus the total number of transfer procedures in which more than one embryo was transferred. Transfer procedures in which only one embryo was transferred but no embryos were cryopreserved were excluded from the calculation of eSET percentages. The average number of embryos transferred for three age groups (<35 years, 35–37 years, and >37 years) was calculated by dividing the total number of embryos transferred by the total number of embryo-transfer procedures performed in that age group.

The contribution of ART to all infants born in a particular reporting area was used as a second measure of ART use. The contribution of ART to an outcome (e.g., preterm or low birthweight infant) was calculated by dividing the total number of outcomes among ART-conceived infants by the total number of outcomes among all infants born. The percentage of infants (ART conceived and all infants) born in the reporting area was calculated by plurality (singleton, multiple, twin, and triplet and higher-order birth) by dividing the number of infants (ART conceived and all infants) in each plurality group by the total number of infants born (ART conceived and all infants). The percentage of infants with low birthweight and preterm delivery was also calculated for each plurality group (singleton, twin, and triplet and higher-order births) for both ART-conceived infants and all infants by dividing the number of low birthweight or preterm infants in each plurality group by the total number of infants in that plurality group.

### Content of This Report

This report provides information on U.S. ART procedures performed in 2014 and compares infant outcomes for ART-conceived infants born in 2014 (resulting from ART procedures performed in 2013 and 2014) with outcomes for all infants born in 2014 in the United States. Specifically, this report provides data on the number and outcomes of all ART procedures performed among patients residing in 52 reporting areas (the 50 states, the District of Columbia, and Puerto Rico) in 2014. For each of these reporting areas, data are presented on the number of ART procedures and embryo transfers performed; the resulting number of pregnancies, live-birth deliveries (overall, singleton, and multiple), and live-born infants; and the number of ART procedures in relation to the number of women in the reproductive age group (15–44 years) (*23*).* Data are also presented on the number of embryo-transfer procedures performed, the average number of embryos transferred, and the percentage of eSET procedures performed among women who used fresh embryos from their own eggs, by age group.

For each reporting area, the proportions of singletons and multiple-birth (including twin and triplet and higher-order) infants resulting from ART are compared with the respective proportions among all infants born in that location in 2014. Infants born in the reporting area during that year include those who were conceived naturally and those resulting from ART and other infertility treatments. To accurately assess the proportion of ART births among overall U.S. births in 2014, ART births were aggregated from two reporting years: 1) infants conceived with ART procedures performed in 2013 and born in 2014 (69.0% of the live-birth deliveries reported to the ART surveillance system for 2014) and 2) infants conceived with ART procedures performed in 2014 and born in 2014 (31.0% of the live-birth deliveries reported to the ART surveillance system for 2014). Data on the total number of live-birth and multiple-birth infants in each reporting area in 2014 were obtained from U.S. natality files (*24*). The report presents the number and percentage of select adverse perinatal outcomes (low birthweight, very low birthweight, preterm delivery, and very preterm delivery) among ART-conceived infants and all infants and the contribution of ART to these outcomes. The percentage of adverse perinatal outcomes is reported for singleton, twin, and triplet and higher-order infants for ART-conceived infants and all infants.

## Results

### Overview of Fertility Clinics

**FIGURE 1:**
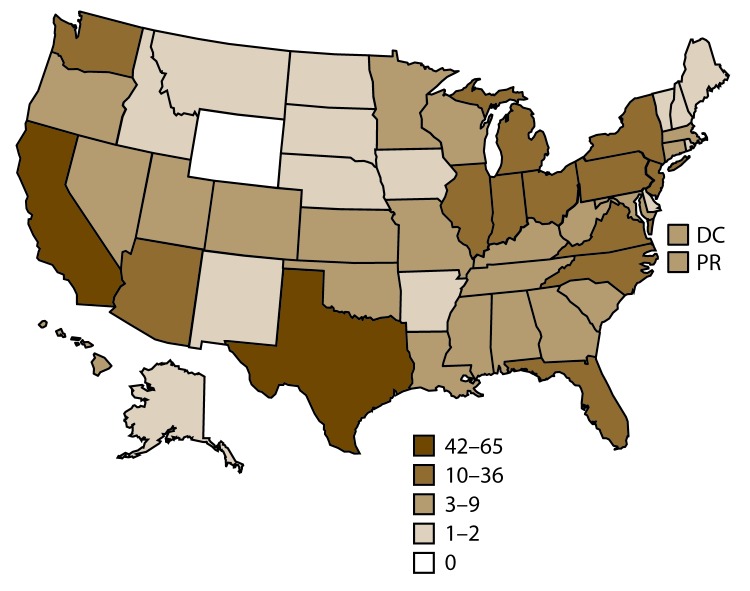
Location and number* of assisted reproductive technology clinics — United States and Puerto Rico, 2014 **Abbreviations:** DC = District of Columbia; PR = Puerto Rico. * In 2014, of the 498 clinics in the United States, 458 (92.2%) submitted data.

Of 498 fertility clinics in the United States that performed ART procedures in 2014, a total of 458 (92.2%) provided data to CDC, with the majority located in or near major cities in the United States. The number of fertility clinics performing ART procedures varied by reporting area. The reporting areas with the largest number of fertility clinics providing data for 2014 were California (65), Texas (42), New York (36), Florida (28), and Illinois (26) (Figure 1).

### Number and Type of ART Procedures

The number, type, and outcome of ART procedures performed in 2014 are provided according to patient’s residence for all 52 reporting areas (Table 1). Residency data are missing for approximately 0.6% of procedures performed and 0.7% of live-birth deliveries; however, they are included in the totals. Approximately 16.6% of ART procedures were conducted in reporting areas other than the patient’s state of residence. Non-U.S. residents accounted for approximately 2.7% of ART procedures, 3.1% of ART live-birth deliveries, and 3.1% of ART infants born.

**TABLE 1:** Number* and outcomes of assisted reproductive technology procedures, by female patient’s reporting area of residence^†^ at time of treatment — United States and Puerto Rico, 2014

Patient’s reporting area of residence	No. of ART clinics^§^	No. of ART procedures performed	No. of ART embryo transfer procedures^¶^	No. of ART pregnancies	No. of ART live-birth deliveries	No. of ART singleton live-birth deliveries	No. of ART multiple live-birth deliveries	No. of ART live-born infants	ART procedures per 1 million women aged 15–44 yrs**
Alabama	6	1,060	849	431	361	262	99	462	1,102.9
Alaska	1	198	176	80	67	50	17	86	1,344.1
Arizona	11	2,116	1,808	972	794	605	189	993	1,617.7
Arkansas	1	457	388	187	165	118	47	212	795.1
California^††^	65	21,018	17,429	8,972	7,230	5,700	1,530	8,793	2,607.2
Colorado	8	1,889	1,578	1,048	856	683	173	1,031	1,741.6
Connecticut	9	3,554	2,680	1,316	1,077	849	228	1,311	5,194.7
Delaware	2	646	520	283	239	220	19	260	3,580.9
District of Columbia	3	1,183	932	398	316	280	36	353	6,662.2
Florida	28	7,559	6,191	2,950	2,374	1,770	604	3,002	2,050.1
Georgia^††^	8	3,640	3,065	1,579	1,293	1,022	271	1,574	1,729.6
Hawaii^††^	5	1,119	847	486	407	291	116	527	4,179.9
Idaho	1	455	397	218	177	104	73	250	1,445.1
Illinois	26	10,671	8,476	4,096	3,317	2,542	775	4,098	4,109.8
Indiana	10	1,950	1,632	790	634	442	192	832	1,505.3
Iowa	2	1,217	1,007	634	512	394	118	630	2,078.1
Kansas	4	1,013	762	386	327	244	83	415	1,807.9
Kentucky	5	1,177	1,038	480	378	259	119	497	1,378.9
Louisiana	5	1,248	965	492	418	309	109	532	1,323.9
Maine	1	382	337	148	111	91	20	130	1,633.7
Maryland	7	5,590	4,519	2,200	1,720	1,481	239	1,965	4,650.9
Massachusetts	8	9,209	7,904	3,618	2,927	2,446	481	3,414	6,725.8
Michigan	12	3,616	3,006	1,454	1,164	851	313	1,484	1,915.5
Minnesota	4	2,012	1,775	993	853	663	190	1,045	1,910.1
Mississippi	3	593	482	229	194	134	60	254	986.3
Missouri	8	2,111	1,811	881	712	549	163	881	1,794.8
Montana	1	258	207	123	101	76	25	125	1,389.6
Nebraska	2	786	621	353	303	208	95	401	2,158.9
Nevada	5	1,246	957	544	449	341	108	560	2,200.4
New Hampshire	1	735	620	285	245	197	48	294	3,016.9
New Jersey	19	8,863	6,914	3,735	3,068	2,430	638	3,708	5,143.3
New Mexico	1	280	242	123	107	88	19	127	704.3
New York	36	20,814	16,212	6,982	5,447	4,349	1,098	6,558	5,144.6
North Carolina	12	3,693	2,903	1,542	1,261	968	293	1,556	1,862.4
North Dakota	1	311	252	128	99	76	23	123	2,157.8
Ohio	12	3,732	3,226	1,577	1,313	949	364	1,687	1,687.5
Oklahoma	3	815	696	351	309	206	103	415	1,068.6
Oregon	4	1,304	1,118	683	597	413	184	787	1,680.2
Pennsylvania	18	6,521	5,282	2,532	2,004	1,624	380	2,389	2,709.1
Puerto Rico	3	260	230	103	77	51	26	104	363.7
Rhode Island	1	701	599	266	210	165	45	256	3,336.3
South Carolina	5	1,394	1,175	589	483	361	122	608	1,477.5
South Dakota	1	266	217	95	78	62	16	96	1,684.7
Tennessee	9	1,525	1,264	660	568	416	152	724	1,177.0
Texas	42	12,750	10,223	5,406	4,471	3,341	1,130	5,636	2,254.8
Utah	3	1,492	1,309	809	668	471	197	870	2,333.5
Vermont	2	210	176	79	64	52	12	76	1,810.6
Virginia	13	5,904	4,781	2,238	1,795	1,493	302	2,103	3,505.7
Washington	12	3,261	2,620	1,414	1,161	946	215	1,381	2,331.8
West Virginia	3	298	259	128	107	82	25	134	885.0
Wisconsin	7	1,759	1,473	759	650	489	161	819	1,617.8
Wyoming	0	124	107	62	52	41	11	64	1,118.9
Nonresident	NA	4,583	3,772	2,101	1,718	1,290	428	2,150	—^§§^
**Total**	**458**	**169,568**	**138,029**	**68,988**	**56,028**	**43,544**	**12,484**	**68,782**	**2,646.5**

**Abbreviations:** ART = assisted reproductive technology; NA = not applicable.

* Excludes 35,406 egg/embryo-freezing and embryo-banking procedures, 3,596 oocyte thaw procedures, and 34 procedures performed in territories not included in this report.

^†^ In cases of missing residency data (0.6%), the patient’s residence was assigned as the location in which the ART procedure was performed.

^§^ The ART procedures and outcomes by patient's reporting area of residence do not necessarily reflect the procedures and outcomes of the ART clinics within the reporting area, as some patients seek treatment at a clinic in a location other than their area of residence.

^¶^ Embryo transfer procedures include all procedures performed in which an attempt was made to transfer at least one embryo.

** On the basis of U.S. Census Bureau estimates (*23*).

^††^ In three states, >2% of residency information was missing for procedures performed: California (2.7%), Georgia (2.1%), and Hawaii (2.1%). Overall, residency information was missing for 963 (0.6%) procedures performed and 366 (0.7%) live-birth deliveries.

^§§^ Non-U.S. residents excluded from rate because the appropriate denominators were not available.

**FIGURE 2:**
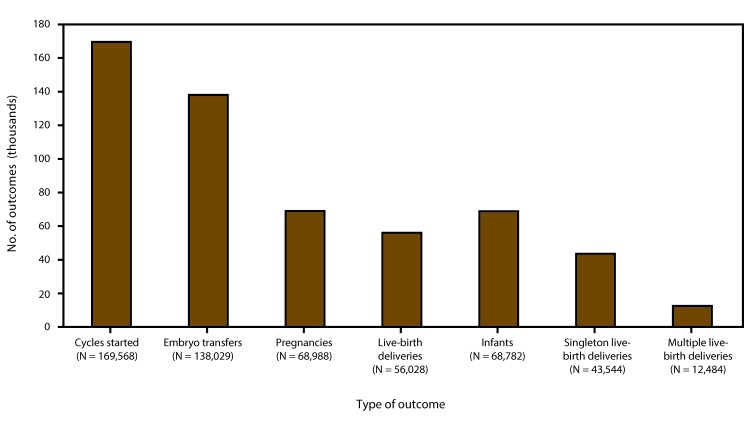
Number of outcomes of assisted reproductive technology procedures, by type of outcome — United States and Puerto Rico, 2014

In 2014, a total of 208,604 ART procedures were reported to CDC (*1*). This report includes data for 169,568 ART procedures performed in the United States (including Puerto Rico) with the intent to transfer at least one embryo (Table 1). It excludes 35,406 egg/embryo-freezing and embryo-banking procedures that did not result in an embryo transfer; 3,596 oocyte-thaw procedures; and 34 procedures that were performed in the remaining territories. Of 169,568 procedures performed in the reporting areas, a total of 138,029 (81.4%) progressed to embryo transfer (Table 1). Of 138,029 ART procedures that progressed to the embryo-transfer stage, 68,988 (50.0%) resulted in a pregnancy and 56,028 (40.6%) in a live-birth delivery. The 56,028 live-birth deliveries included 43,544 singleton live-birth deliveries (77.7%) and 12,484 multiple live-birth deliveries (22.3%) and resulted in 68,782 live-born infants (Table 1) (Figure 2).

Six reporting areas with the largest number of ART procedures (California, Illinois, Massachusetts, New Jersey, New York, and Texas) accounted for 49.1% (83,325 of 169,568) of all ART procedures, 48.6% (67,158 of 138,029) of all embryo-transfer procedures, 46.8% (32,207 of 68,782) of all infants born from ART, and 45.3% (5,652 of 12,484) of all ART multiple live-birth deliveries in the United States (Table 1); however, these six reporting areas accounted for only 36.7% of all U.S. births (*24*).

**FIGURE 3:**
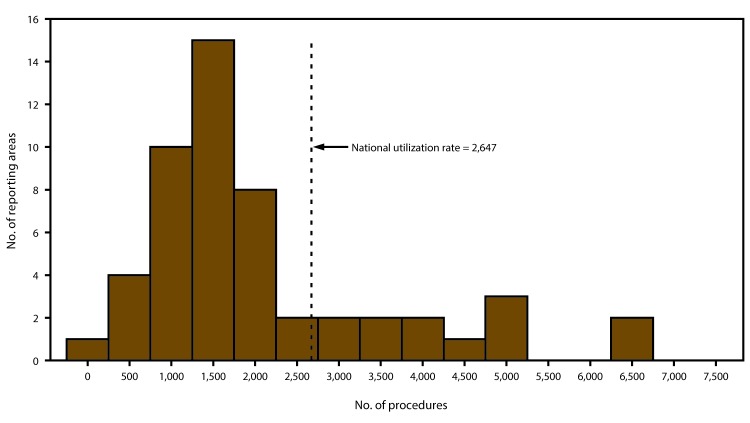
Number of reporting areas by number of assisted reproductive technology procedures performed among women of reproductive age (15–44 years)* — United States and Puerto Rico, 2014 * Per 1 million women aged 15–44 years.

The number of ART procedures per 1 million women of reproductive age (15–44 years) varied from 364 in Puerto Rico to 6,726 in Massachusetts, with an overall national rate of 2,647. Thirteen reporting areas (Connecticut, Delaware, Hawaii, Illinois, Maryland, Massachusetts, New Hampshire, New Jersey, New York, Pennsylvania, Rhode Island, Virginia, and the District of Columbia) had rates higher than the national rate. Of these reporting areas, Massachusetts (6,726) and the District of Columbia (6,662) had rates exceeding twice the national rate, while Connecticut (5,195), Hawaii (4,180), Illinois (4,110), Maryland (4,651), New Jersey (5,143) and New York (5,145) had rates exceeding 1.5 times the national rate (Table 1) (Figure 3).

### Embryo Transfer and Patient’s Age

The number of embryo-transfer procedures performed, the average number of embryos transferred per procedure, and the percentage of eSET procedures performed among women who used fresh embryos from their own eggs are provided by reporting area and age group (Table 2). Overall, the number of embryo-transfer procedures performed was 30,887 among women aged <35 years, 14,647 among women aged 35–37 years, and 21,504 among women aged >37 years. Nationally, the average number of embryos transferred per procedure was 1.7 among women aged <35 years (range: 1.3 in Delaware to 2.1 in Puerto Rico), 1.9 among women aged 35–37 years (range: 1.5 in Maine to 2.3 in Hawaii), and 2.3 among women aged >37 years (range: 1.9 in Nevada to 3.0 in Montana). Nationally, percentage of eSET was 28.5% among women aged <35 years (range: 4.3% in Puerto Rico to 67.9% in Delaware), 16.7% among women aged 35–37 years (range: 0% in South Dakota to 39.5% in Maine), and 4.7% among women aged >37 years (range: 0% in 9 reporting areas to 12.5% in Delaware and Idaho). Among women aged <35 years, eSET percentage exceeded 1.5 times the national rate in six reporting areas (Delaware, the District of Columbia, Maine, Massachusetts, Maryland, and New Hampshire).

**TABLE 2:** Number of assisted reproductive technology embryo transfer procedures* among patients who used fresh embryos from their own eggs, by female patient’s age group and reporting area of residence^†^ at time of treatment — United States and Puerto Rico, 2014

Patient’s reporting area of residence	<35 yrs			35–37 yrs			>37 yrs		
	No. of embryo transfer procedures	Average no. of embryos transferred	eSET^§^ (%)	No. of embryo transfer procedures	Average no. of embryos transferred	eSET^§^ (%)	No. of embryo transfer procedures	Average no. of embryos transferred	eSET^§^ (%)
Alabama	297	1.8	16.4	98	2.1	6.6	79	2.7	0.0
Alaska	47	1.8	26.8	27	1.9	18.2	15	—^¶^	—^¶^
Arizona	331	1.8	24.5	148	2.1	6.7	178	2.3	2.7
Arkansas	136	1.8	14.8	40	2.0	2.9	27	2.0	0.0
California**	2,543	1.7	29.7	1,650	1.9	19.0	2,859	2.6	3.8
Colorado	200	1.8	22.6	79	2.0	8.6	50	2.3	4.9
Connecticut	731	1.6	32.1	348	1.8	14.1	563	2.3	1.9
Delaware	89	1.3	67.9	23	1.7	36.8	34	2.1	12.5
District of Columbia	110	1.4	60.4	107	1.6	35.4	261	2.1	8.2
Florida	1,477	1.7	23.7	715	1.9	9.1	1,031	2.3	2.5
Georgia**	697	1.6	35.5	306	1.9	11.5	345	2.4	3.0
Hawaii**	114	1.9	17.4	87	2.3	4.9	179	2.7	2.6
Idaho	120	1.9	13.4	46	2.1	2.4	30	2.0	12.5
Illinois	2,091	1.7	27.2	945	1.9	13.1	1,388	2.3	2.7
Indiana	620	1.9	10.4	189	2.0	9.0	175	2.2	2.1
Iowa	310	1.6	39.6	80	1.7	26.5	77	2.3	4.3
Kansas	239	1.7	23.3	61	2.0	3.6	53	2.1	2.1
Kentucky	372	1.9	18.3	132	1.9	8.3	106	2.3	1.1
Louisiana	293	1.9	11.2	69	2.0	4.8	97	2.2	6.2
Maine	91	1.4	58.7	46	1.5	39.5	60	2.0	8.3
Maryland	1,121	1.4	51.8	592	1.6	35.1	918	2.1	8.4
Massachusetts	1,930	1.4	51.5	1,137	1.6	32.1	1,952	2.5	7.0
Michigan	861	1.9	15.0	308	2.0	10.5	370	2.3	4.1
Minnesota	571	1.7	30.2	175	1.8	17.3	194	2.0	7.8
Mississippi	120	1.8	22.4	43	2.0	5.3	52	2.1	0.0
Missouri	601	1.8	13.5	183	2.0	6.3	154	2.6	0.0
Montana	70	1.6	36.9	16	—^¶^	—^¶^	28	3.0	0.0
Nebraska	211	1.8	12.8	72	1.8	7.0	46	2.3	0.0
Nevada	191	1.7	24.4	73	1.7	16.9	92	1.9	10.0
New Hampshire	189	1.4	51.0	99	1.6	29.9	93	2.4	3.8
New Jersey	1,310	1.6	34.7	699	1.8	19.1	1,054	2.2	8.2
New Mexico	31	1.8	17.9	8	—^¶^	—^¶^	13	—^¶^	—^¶^
New York	3,075	1.7	30.0	1,835	1.9	15.5	3,883	2.3	5.2
North Carolina	621	1.7	28.1	280	1.8	15.9	310	2.4	3.3
North Dakota	89	1.8	17.2	22	1.9	9.5	25	2.2	4.5
Ohio	1,087	1.9	14.9	442	2.0	6.5	393	2.4	2.9
Oklahoma	303	1.9	6.2	76	1.9	3.2	62	2.4	0.0
Oregon	248	1.9	15.2	123	1.8	15.8	89	2.2	9.0
Pennsylvania	1,357	1.7	33.9	629	1.9	16.6	743	2.3	5.9
Puerto Rico	74	2.1	4.3	48	2.2	4.7	59	2.4	0.0
Rhode Island	162	1.6	34.5	96	1.9	11.4	138	2.6	4.1
South Carolina	265	1.9	14.7	120	2.0	6.4	154	2.4	4.5
South Dakota	68	1.7	30.2	21	1.9	0	14	—^¶^	—^¶^
Tennessee	316	1.8	22.8	115	2.0	6.7	111	2.4	2.2
Texas	2,128	1.8	21.1	933	2.0	8.4	1,139	2.3	1.6
Utah	478	1.8	18.0	137	2.0	7.1	118	2.2	3.1
Vermont	50	1.7	21.4	34	1.8	19.4	33	2.3	3.2
Virginia	1,038	1.5	40.3	540	1.7	31.1	894	2.1	7.2
Washington	540	1.6	38.0	235	1.7	24.8	297	2.1	6.4
West Virginia	92	1.8	18.5	30	1.9	8.3	27	2.4	0.0
Wisconsin	429	1.7	27.1	134	1.8	18.5	125	2.2	4.6
Wyoming	37	1.8	13.9	11	—^¶^	—^¶^	6	—^¶^	—^¶^
Nonresident	316	1.8	22.8	185	1.7	19.9	311	2.2	8.9
**Total**	**30,887**	**1.7**	**28.5**	**14,647**	**1.9**	**16.7**	**21,504**	**2.3**	**4.7**

**Abbreviation:** eSET = elective single-embryo transfer.

* Includes all procedures in which at least one embryo was transferred.

^†^ In cases of missing residency data (0.6%), the patient's residence was assigned as the location in which the assisted reproductive technology procedure was performed.

^§^ A procedure in which one embryo, selected from a larger number of available embryos, is placed in the uterus. A cycle in which only one embryo is available is not defined as eSET.

^¶^ Estimates on the basis of N <20 in the denominator have been suppressed because such rates are considered unstable.

** In three states, >2% of residency information was missing for procedures performed: California (2.7%), Georgia (2.1%), and Hawaii (2.1%). Overall, residency information was missing for 963 (0.6%) procedures performed and 366 (0.7%) live-birth deliveries.

### Singleton and Multiple-Birth Infants

Among 4,022,510 infants born in the United States and Puerto Rico in 2014 (*21*), a total of 65,296 (1.6%) were conceived with ART procedures performed in 2013 and 2014 (Table 3). California, Texas, and New York had the highest total number of all infants born (502,879, 399,766, and 238,773, respectively), as well as ART-conceived infants born (8,528, 5,268, and 6,492, respectively). The contribution of ART to all infants born was highest in Massachusetts (4.7%), followed by the District of Columbia (3.8%), New Jersey (3.7%), and Connecticut (3.6%). Although singletons accounted for 96.5% of all infants born in 2014 (range: 95.6% in New Jersey to 97.8% in Puerto Rico), singletons accounted for only 60.6% of all ART-conceived infants (range: 44.4% in Puerto Rico to 88.5% in Delaware).

**TABLE 3:** Number, proportion, and percentage of infants born with the use of assisted reproductive technology, by female patient's reporting area of residence* at time of treatment — United States and Puerto Rico, 2014^†^

Patient’s reporting area of residence	Total no. of infants born^§^	No. of ART infants born	Proportion of ART infants among all infants (%)	Singleton infants among ART infants	Singleton infants among all infants^§^	Proportion of ART singleton infants among all singleton infants (%)
				No. (%)	No. (%)	
Alabama	59,422	466	0.8	231 (49.6)	57,307 (96.4)	0.4
Alaska	11,392	56	0.5	36 (64.3)	11,031 (96.8)	0.3
Arizona	86,887	1,030	1.2	616 (59.8)	84,240 (97.0)	0.7
Arkansas	38,511	196	0.5	118 (60.2)	37,358 (97.0)	0.3
California^¶^	502,879	8,528	1.7	5,389 (63.2)	486,558 (96.8)	1.1
Colorado	65,830	993	1.5	580 (58.4)	63,748 (96.8)	0.9
Connecticut	36,285	1,321	3.6	829 (62.8)	34,715 (95.7)	2.4
Delaware	10,972	226	2.1	200 (88.5)	10,654 (97.1)	1.9
District of Columbia	9,509	361	3.8	283 (78.4)	9,153 (96.3)	3.1
Florida	219,991	2,900	1.3	1,641 (56.6)	212,467 (96.6)	0.8
Georgia^¶^	130,946	1,487	1.1	886 (59.6)	126,403 (96.5)	0.7
Hawaii^¶^	18,550	447	2.4	230 (51.5)	17,969 (96.9)	1.3
Idaho	22,876	218	1.0	116 (53.2)	22,186 (97.0)	0.5
Illinois	158,556	4,074	2.6	2,459 (60.4)	152,251 (96.0)	1.6
Indiana	84,080	814	1.0	431 (52.9)	81,163 (96.5)	0.5
Iowa	39,687	629	1.6	399 (63.4)	38,256 (96.4)	1.0
Kansas	39,223	361	0.9	216 (59.8)	37,964 (96.8)	0.6
Kentucky	56,170	508	0.9	247 (48.6)	54,304 (96.7)	0.5
Louisiana	64,497	542	0.8	284 (52.4)	62,124 (96.3)	0.5
Maine	12,698	146	1.1	95 (65.1)	12,224 (96.3)	0.8
Maryland	73,921	1,956	2.6	1,423 (72.8)	71,056 (96.1)	2.0
Massachusetts	71,908	3,413	4.7	2,302 (67.4)	68,839 (95.7)	3.3
Michigan	11,4375	1,503	1.3	830 (55.2)	110,146 (96.3)	0.8
Minnesota	69,904	1,172	1.7	689 (58.8)	67,312 (96.3)	1.0
Mississippi	38,736	248	0.6	145 (58.5)	37,390 (96.5)	0.4
Missouri	75,360	870	1.2	492 (56.6)	72,727 (96.5)	0.7
Montana	12,432	128	1.0	65 (50.8)	12,004 (96.6)	0.5
Nebraska	26,794	351	1.3	168 (47.9)	25,859 (96.5)	0.6
Nevada	35,861	559	1.6	332 (59.4)	34,726 (96.8)	1.0
New Hampshire	12,302	295	2.4	184 (62.4)	11,844 (96.3)	1.6
New Jersey	103,305	3,786	3.7	2,384 (63.0)	98,805 (95.6)	2.4
New Mexico	26,052	129	0.5	81 (62.8)	25,445 (97.7)	0.3
New York	238,773	6,492	2.7	4,175 (64.3)	229,570 (96.1)	1.8
North Carolina	120,975	1,559	1.3	898 (57.6)	116,626 (96.4)	0.8
North Dakota	11,359	142	1.3	77 (54.2)	10,977 (96.6)	0.7
Ohio	139,467	1,672	1.2	882 (52.8)	134,327 (96.3)	0.7
Oklahoma	53,339	419	0.8	213 (50.8)	51,681 (96.9)	0.4
Oregon	45,556	672	1.5	338 (50.3)	43,941 (96.5)	0.8
Pennsylvania	142,268	2,442	1.7	1,540 (63.1)	136,985 (96.3)	1.1
Puerto Rico	34,434	133	0.4	59 (44.4)	33,663 (97.8)	0.2
Rhode Island	10,823	232	2.1	147 (63.4)	10,453 (96.6)	1.4
South Carolina	57,627	548	1.0	309 (56.4)	55,548 (96.4)	0.6
South Dakota	12,283	126	1.0	68 (54.0)	11,882 (96.7)	0.6
Tennessee	81,602	634	0.8	362 (57.1)	78,919 (96.7)	0.5
Texas	399,766	5,268	1.3	2,892 (54.9)	386,674 (96.7)	0.7
Utah	51,154	730	1.4	349 (47.8)	49,287 (96.4)	0.7
Vermont	6,130	107	1.7	56 (52.3)	5,874 (95.8)	1.0
Virginia	103,300	2,163	2.1	1,467 (67.8)	99,469 (96.3)	1.5
Washington	88,585	1,299	1.5	808 (62.2)	85,847 (96.9)	0.9
West Virginia	20,301	101	0.5	64 (63.4)	19,623 (96.7)	0.3
Wisconsin	67,161	790	1.2	464 (58.7)	64,864 (96.6)	0.7
Wyoming	7,696	54	0.7	33 (61.1)	7,439 (96.7)	0.4
**Total**	**4,022,510**	**65,296**	**1.6**	**39,582 (60.6)**	**3,881,877 (96.5)**	**1.0**

**Abbreviation:** ART = assisted reproductive technology.

* In cases of missing residency data (0.6%), the patient's residence was assigned as the location in which the ART procedure was performed.

^†^ Includes infants conceived from ART procedures performed in 2013 and born in 2014 and infants conceived from ART procedures performed in 2014 and born in 2014. Total ART births exclude nonresidents.

^§^ U.S. births include nonresidents (*24*).

^¶^ In three states, >2% of residency information was missing for procedures performed: California (2.7%), Georgia (2.1%), and Hawaii (2.1%). Overall, residency information was missing for 963 (0.6%) procedures performed and 366 (0.7%) live-birth deliveries.

Nationwide, 39.4% of ART-conceived infants were born in multiple-birth deliveries (range: 11.5% in Delaware to 55.6% in Puerto Rico), compared with only 3.5% of all infants (range: 2.2% in Puerto Rico to 4.4% in New Jersey) (Table 4). ART-conceived twins accounted for approximately 95.3% (24,514 of 25,714) of all ART-conceived infants born in multiple deliveries. ART multiple-birth infants contributed to 18.3% of all multiple-birth infants (range: 5.5% in Alaska and West Virginia to 37.3% in Hawaii). Approximately 37.5% of all ART-conceived infants were twins (range: 11.5% in Delaware to 51.1% in Puerto Rico), compared with 3.4% of all infants (range: 2.2% in New Mexico and Puerto Rico to 4.2% in Connecticut, Massachusetts, and New Jersey). ART-conceived twin infants contributed to 18.0% of all twins born in 2014 (range: 5.2% in some states to 36.2% in Hawaii). Finally, 1.8% of ART-conceived infants were triplets and higher-order multiples (range: 0% in Alaska, Delaware, and Montana to 5.6% in some states), compared with 0.1%–0.2% of all infants. ART triplet and higher-order infants contributed to 26.4% of all triplet and higher-order infants born in 2014 (range: 0% in Alaska, Delaware, and Montana to 65.2% in Hawaii).

**TABLE 4:** Number, percentage, and proportion of multiple-birth, twins, and triplets and higher-order infants born with use of assisted reproductive technology procedure, by female patient’s reporting area of residence* at time of treatment — United States and Puerto Rico, 2014^†^

Patient’s reporting area of residence	Multiple-birth infants among ART infants^§^	Multiple-birth infants among all infants^¶^	Proportion of ART multiple-birth infants among all multiple-birth infants (%)	Twin infants among ART infants^§^	Twin infants among all infants^¶^	Proportion of ART twin infants among all twin infants (%)	Triplets and higher-order infants among ART infants^§^	Triplets and higher-order infants among all infants^¶^	Proportion of ART triplets and higher-order infants among all triplets and higher-order infants (%)
	No. (%)	No. (%)		No. (%)	No. (%)		No. (%)	No. (%)	
Alabama	235 (50.4)	2,115 (3.6)	11.1	229 (49.1)	2,072 (3.5)	11.1	6 (1.3)	43 (0.1)	14.0
Alaska	20 (35.7)	361 (3.2)	5.5	20 (35.7)	— (—)**	—**	0 (0.0)	— (—)**	0.0
Arizona	414 (40.2)	2,647 (3.0)	15.6	392 (38.1)	2,579 (3.0)	15.2	22 (2.1)	68 (0.1)	32.4
Arkansas	78 (39.8)	1,153 (3.0)	6.8	72 (36.7)	1,110 (2.9)	6.5	6 (3.1)	43 (0.1)	14.0
California^¶¶^	3,139 (36.8)	16,321 (3.2)	19.2	2,976 (34.9)	15,832 (3.1)	18.8	163 (1.9)	489 (0.1)	33.3
Colorado	413 (41.6)	2,082 (3.2)	19.8	392 (39.5)	2,019 (3.1)	19.4	21 (2.1)	63 (0.1)	33.3
Connecticut	492 (37.2)	1,570 (4.3)	31.3	474 (35.9)	1,525 (4.2)	31.1	18 (1.4)	45 (0.1)	40.0
Delaware	26 (11.5)	318 (2.9)	8.2	26 (11.5)	— (—)**	—**	0 (0.0)	— (—)**	0.0
District of Columbia	78 (21.6)	356 (3.7)	21.9	72 (19.9)	344 (3.6)	20.9	6 (1.7)	12 (0.1)	—^††^
Florida	1,259 (43.4)	7,524 (3.4)	16.7	1,190 (41.0)	7,307 (3.3)	16.3	69 (2.4)	217 (0.1)	31.8
Georgia^¶¶^	601 (40.4)	4,543 (3.5)	13.2	571 (38.4)	4,411 (3.4)	12.9	30 (2.0)	132 (0.1)	22.7
Hawaii^¶¶^	217 (48.5)	581 (3.1)	37.3	202 (45.2)	558 (3.0)	36.2	15 (3.4)	23 (0.1)	65.2
Idaho	102 (46.8)	690 (3.0)	14.8	— (—)**	678 (3.0)	—**	— (—)**	12 (0.1)	—**^,††^
Illinois	1,615 (39.6)	6,305 (4.0)	25.6	1,552 (38.1)	6,085 (3.8)	25.5	63 (1.5)	220 (0.1)	28.6
Indiana	383 (47.1)	2,917 (3.5)	13.1	359 (44.1)	2,799 (3.3)	12.8	24 (2.9)	118 (0.1)	20.3
Iowa	230 (36.6)	1,431 (3.6)	16.1	— (—)**	1,365 (3.4)	—**	— (—)**	66 (0.2)	—**
Kansas	145 (40.2)	1,259 (3.2)	11.5	130 (36.0)	1,213 (3.1)	10.7	15 (4.2)	46 (0.1)	32.6
Kentucky	261 (51.4)	1,866 (3.3)	14.0	234 (46.1)	1,794 (3.2)	13.0	27 (5.3)	72 (0.1)	37.5
Louisiana	258 (47.6)	2,373 (3.7)	10.9	246 (45.4)	2,295 (3.6)	10.7	12 (2.2)	78 (0.1)	15.4
Maine	51 (34.9)	474 (3.7)	10.8	— (—)**	453 (3.6)	—**	— (—)**	21 (0.2)	—**
Maryland	533 (27.2)	2,865 (3.9)	18.6	516 (26.4)	2,783 (3.8)	18.5	17 (0.9)	82 (0.1)	20.7
Massachusetts	1,111 (32.6)	3,069 (4.3)	36.2	1,066 (31.2)	2,986 (4.2)	35.7	45 (1.3)	83 (0.1)	54.2
Michigan	673 (44.8)	4,229 (3.7)	15.9	643 (42.8)	4,100 (3.6)	15.7	30 (2.0)	129 (0.1)	23.3
Minnesota	483 (41.2)	2,592 (3.7)	18.6	469 (40.0)	2,535 (3.6)	18.5	14 (1.2)	57 (0.1)	24.6
Mississippi	103 (41.5)	1,346 (3.5)	7.7	97 (39.1)	1,316 (3.4)	7.4	6 (2.4)	30 (0.1)	20.0
Missouri	378 (43.4)	2,633 (3.5)	14.4	363 (41.7)	2,567 (3.4)	14.1	15 (1.7)	66 (0.1)	22.7
Montana	63 (49.2)	428 (3.4)	14.7	63 (49.2)	420 (3.4)	15.0	0 (0.0)	8 (0.1)	0.0
Nebraska	183 (52.1)	935 (3.5)	19.6	168 (47.9)	878 (3.3)	19.1	15 (4.3)	57 (0.2)	26.3
Nevada	227 (40.6)	1,135 (3.2)	20.0	— (—)**	1,109 (3.1)	—**	— (—)**	26 (0.1)	—**
New Hampshire	111 (37.6)	458 (3.7)	24.2	— (—)**	452 (3.7)	—**	— (—)**	6 (0.0)	—**^,††^
New Jersey	1,402 (37.0)	4,500 (4.4)	31.2	1,360 (35.9)	4,343 (4.2)	31.3	42 (1.1)	157 (0.2)	26.8
New Mexico	48 (37.2)	607 (2.3)	7.9	42 (32.6)	580 (2.2)	7.2	6 (4.7)	27 (0.1)	22.2
New York	2,317 (35.7)	9,203 (3.9)	25.2	2,198 (33.9)	8,840 (3.7)	24.9	119 (1.8)	363 (0.2)	32.8
North Carolina	661 (42.4)	4,349 (3.6)	15.2	643 (41.2)	4,233 (3.5)	15.2	18 (1.2)	116 (0.1)	15.5
North Dakota	65 (45.8)	382 (3.4)	17.0	— (—)**	360 (3.2)	—**	— (—)**	22 (0.2)	—**
Ohio	790 (47.2)	5,140 (3.7)	15.4	741 (44.3)	4,904 (3.5)	15.1	49 (2.9)	236 (0.2)	20.8
Oklahoma	206 (49.2)	1,658 (3.1)	12.4	189 (45.1)	1,582 (3.0)	11.9	17 (4.1)	76 (0.1)	22.4
Oregon	334 (49.6)	1,615 (3.5)	20.7	328 (48.7)	1,572 (3.5)	20.9	6 (0.9)	43 (0.1)	14.0
Pennsylvania	902 (36.9)	5,283 (3.7)	17.1	875 (35.8)	5,158 (3.6)	17.0	27 (1.1)	125 (0.1)	21.6
Puerto Rico	74 (55.6)	771 (2.2)	9.6	68 (51.1)	750 (2.2)	9.1	6 (4.5)	21 (0.1)	28.6
Rhode Island	85 (36.6)	370 (3.4)	23.0	— (—)**	358 (3.3)	—**	— (—)**	12 (0.1)	—**^,††^
South Carolina	239 (43.6)	2,079 (3.6)	11.5	— (—)**	2,030 (3.5)	—**	— (—)**	49 (0.1)	—**
South Dakota	58 (46.0)	401 (3.3)	14.5	52 (41.3)	389 (3.2)	13.4	6 (4.8)	12 (0.1)	—^††^
Tennessee	272 (42.9)	2,683 (3.3)	10.1	258 (40.7)	2,596 (3.2)	9.9	14 (2.2)	87 (0.1)	16.1
Texas	2,376 (45.1)	13,092 (3.3)	18.1	2,238 (42.5)	12,600 (3.2)	17.8	138 (2.6)	492 (0.1)	28.0
Utah	381 (52.2)	1,867 (3.6)	20.4	365 (50.0)	1,797 (3.5)	20.3	16 (2.2)	70 (0.1)	22.9
Vermont	51 (47.7)	256 (4.2)	19.9	— (—)**	250 (4.1)	—**	— (—)**	6 (0.1)	—**^,††^
Virginia	696 (32.2)	3,831 (3.7)	18.2	662 (30.6)	3,682 (3.6)	18.0	34 (1.6)	149 (0.1)	22.8
Washington	491 (37.7)	2,738 (3.1)	17.9	470 (36.0)	2,660 (3.0)	17.7	21 (1.6)	78 (0.1)	26.9
West Virginia	37 (36.6)	678 (3.3)	5.5	— (—)**	657 (3.2)	—**	— (—)**	21 (0.1)	—**
Wisconsin	326 (41.3)	2,297 (3.4)	14.2	317 (40.1)	2,236 (3.3)	14.2	9 (1.1)	61 (0.1)	14.8
Wyoming	21 (38.9)	257 (3.3)	8.2	— (—)**	251 (3.3)	—**	— (—)**	6 (0.1)	—**^,††^
**Total**	**25,714 (39.4)**	**140,633 (3.5)**	**18.3**	**24,514 (37.5)**	**136,086 (3.4)**	**18.0**	**1,200 (1.8)**	**4,547 (0.1)**	**26.4**

**Abbreviation:** ART = assisted reproductive technology.

* In cases of missing residency data (0.6%), the patient's residence was assigned as the location in which the ART procedure was performed.

^†^ ART totals include infants conceived from ART procedures performed in 2013 and born in 2014 and infants conceived from ART procedures performed in 2014 and born in 2014. Total ART births exclude nonresidents.

^§^ Includes only the number of infants live-born in a multiple-birth delivery. For example, if three infants were born in a live-birth delivery and one of the three infants was stillborn, the total number of live-born infants would be two. However, the two infants still would be counted as triplets.

^¶^ U.S. births include nonresidents (*24*).

** To protect confidentiality, cells with values from 1–4 are suppressed, as are data that can be used to derive cell values of 1–4. These values are included in totals.

^††^Estimates on the basis of N <20 in the denominator have been suppressed because such rates are considered unstable.

^¶¶^ In three states, >2% of residency information was missing for procedures performed: California (2.7%), Georgia (2.1%), and Hawaii (2.1%). Overall, residency information was missing for 963 (0.6%) procedures performed and 366 (0.7%) live-birth deliveries.

### Adverse Perinatal Outcomes

Nationally, ART-conceived infants contributed to approximately 5.5% of all low birthweight infants (range: 1.2% in West Virginia to 14.2% in Massachusetts) and 5.6% of all very low birthweight infants (range: 0.9% in some states to 14.0% in Hawaii) (Table 5). In four reporting areas (Connecticut, Hawaii, Massachusetts, and New Jersey), >10% of all low birthweight and all very low birthweight infants born were conceived with ART. In all reporting areas, the percentage of low birthweight and very low birthweight infants was higher among infants conceived with ART than among all infants. Among ART-conceived infants, 27.8% had low birthweight (range: 10.6% in Delaware to 44.4% in Puerto Rico), compared with 8.0% among all infants (range: 5.9% in Alaska to 11.3% in Mississippi). Approximately 4.9% of ART-conceived infants had very low birthweight (range: 0.9% in some states to 9.6% in Louisiana), compared with 1.4% among all infants (range: 0.9% in Alaska to 2.1% in the District of Columbia and Mississippi).

**TABLE 5:** Number, percentage, and proportion of infants born with use of assisted reproductive technology,* by low birthweight category and female patient’s reporting area of residence^†^ at time of treatment — United States and Puerto Rico, 2014

Patient’s reporting area of residence	<2,500 g (LBW)			<1,500 g (VLBW)		
	ART infants	All infants^§^	Proportion of ART LBW infants among all LBW infants (%)	ART infants	All infants^§^	Proportion of ART VLBW infants among all VLBW infants (%)
	No. (%)	No. (%)		No. (%)	No. (%)	
Alabama	162 (35.2)	5,989 (10.1)	2.7	25 (5.4)	1,135 (1.9)	2.2
Alaska	16 (29.6)	672 (5.9)	2.4	— (—)^¶^	101 (0.9)	—^¶^
Arizona	283 (28.3)	6,086 (7.0)	4.7	55 (5.5)	1,004 (1.2)	5.5
Arkansas	61 (31.1)	3,432 (8.9)	1.8	11 (5.6)	558 (1.4)	2.0
California**	2,091 (25.2)	33,586 (6.7)	6.2	360 (4.3)	5,727 (1.1)	6.3
Colorado	334 (34.2)	5,769 (8.8)	5.8	45 (4.6)	799 (1.2)	5.6
Connecticut	325 (24.8)	2,763 (7.6)	11.8	67 (5.1)	499 (1.4)	13.4
Delaware	24 (10.6)	908 (8.3)	2.6	8 (3.5)	194 (1.8)	4.1
District of Columbia	59 (16.4)	934 (9.8)	6.3	8 (2.2)	198 (2.1)	4.0
Florida	831 (29.4)	19,065 (8.7)	4.4	160 (5.7)	3,501 (1.6)	4.6
Georgia**	445 (30.2)	12,385 (9.5)	3.6	87 (5.9)	2,321 (1.8)	3.7
Hawaii**	165 (38.6)	1,462 (7.9)	11.3	34 (7.9)	243 (1.3)	14.0
Idaho	74 (33.9)	1,471 (6.4)	5.0	— (—)^¶^	227 (1.0)	—^¶^
Illinois	1,119 (27.7)	12,929 (8.2)	8.7	186 (4.6)	2,409 (1.5)	7.7
Indiana	242 (29.8)	6,715 (8.0)	3.6	39 (4.8)	1,146 (1.4)	3.4
Iowa	156 (24.8)	2,675 (6.7)	5.8	24 (3.8)	460 (1.2)	5.2
Kansas	108 (30.5)	2,759 (7.0)	3.9	17 (4.8)	493 (1.3)	3.4
Kentucky	179 (35.9)	4,922 (8.8)	3.6	42 (8.4)	819 (1.5)	5.1
Louisiana	195 (36.0)	6,786 (10.5)	2.9	52 (9.6)	1,230 (1.9)	4.2
Maine	34 (23.6)	960 (7.6)	3.5	7 (4.9)	157 (1.2)	4.5
Maryland	471 (24.2)	6,345 (8.6)	7.4	78 (4.0)	1,186 (1.6)	6.6
Massachusetts	761 (22.6)	5,351 (7.4)	14.2	113 (3.4)	860 (1.2)	13.1
Michigan	458 (30.6)	9,545 (8.3)	4.8	89 (5.9)	1,688 (1.5)	5.3
Minnesota	308 (26.5)	4,595 (6.6)	6.7	56 (4.8)	790 (1.1)	7.1
Mississippi	69 (28.3)	4,374 (11.3)	1.6	13 (5.3)	804 (2.1)	1.6
Missouri	259 (30.9)	6,163 (8.2)	4.2	43 (5.1)	1,022 (1.4)	4.2
Montana	45 (35.4)	920 (7.4)	4.9	12 (9.4)	152 (1.2)	7.9
Nebraska	109 (31.2)	1,775 (6.6)	6.1	18 (5.2)	312 (1.2)	5.8
Nevada	154 (28.5)	2,972 (8.3)	5.2	20 (3.7)	509 (1.4)	3.9
New Hampshire	82 (28.0)	852 (6.9)	9.6	16 (5.5)	130 (1.1)	12.3
New Jersey	1,013 (26.9)	8,315 (8.0)	12.2	178 (4.7)	1,519 (1.5)	11.7
New Mexico	40 (31.3)	2,282 (8.8)	1.8	12 (9.4)	349 (1.3)	3.4
New York	1,676 (27.0)	18,722 (7.8)	9.0	304 (4.9)	3,298 (1.4)	9.2
North Carolina	425 (27.3)	10,720 (8.9)	4.0	68 (4.4)	1,997 (1.7)	3.4
North Dakota	43 (30.3)	704 (6.2)	6.1	13 (9.2)	116 (1.0)	11.2
Ohio	531 (31.9)	11,800 (8.5)	4.5	100 (6.0)	2,185 (1.6)	4.6
Oklahoma	143 (34.1)	4,238 (7.9)	3.4	24 (5.7)	766 (1.4)	3.1
Oregon	191 (28.6)	2,842 (6.2)	6.7	23 (3.4)	455 (1.0)	5.1
Pennsylvania	628 (25.8)	11,713 (8.2)	5.4	100 (4.1)	2,006 (1.4)	5.0
Puerto Rico	59 (44.4)	3,713 (10.8)	1.6	11 (8.3)	502 (1.5)	2.2
Rhode Island	47 (20.4)	765 (7.1)	6.1	11 (4.8)	150 (1.4)	7.3
South Carolina	165 (30.5)	5,435 (9.4)	3.0	32 (5.9)	1,016 (1.8)	3.1
South Dakota	33 (26.2)	804 (6.5)	4.1	5 (4.0)	125 (1.0)	4.0
Tennessee	202 (31.9)	7,297 (8.9)	2.8	39 (6.2)	1,260 (1.5)	3.1
Texas	1,729 (33.3)	32,744 (8.2)	5.3	353 (6.8)	5,722 (1.4)	6.2
Utah	261 (36.0)	3,572 (7.0)	7.3	37 (5.1)	552 (1.1)	6.7
Vermont	35 (33.3)	432 (7.0)	8.1	9 (8.6)	65 (1.1)	13.8
Virginia	497 (23.2)	8,130 (7.9)	6.1	89 (4.2)	1,547 (1.5)	5.8
Washington	302 (23.4)	5,705 (6.4)	5.3	31 (2.4)	888 (1.0)	3.5
West Virginia	23 (23.2)	1,852 (9.1)	1.2	5 (5.1)	278 (1.4)	1.8
Wisconsin	198 (25.3)	4,911 (7.3)	4.0	38 (4.9)	876 (1.3)	4.3
Wyoming	15 (27.8)	704 (9.1)	2.1	— (—)^¶^	103 (1.3)	—^¶^
**Total**	**17,875 (27.8)**	**322,560 (8.0)**	**5.5**	**3,174 (4.9)**	**56,449 (1.4)**	**5.6**

**Abbreviations:** ART = assisted reproductive technology; LBW = low birthweight; VLBW = very low birthweight.

* ART totals include infants conceived from ART procedures performed in 2013 and born in 2014 and infants conceived from ART procedures performed in 2014 and born in 2014. Total ART infants exclude nonresidents and include only infants with birthweight data available.

^†^ In cases of missing residency data (0.6%), the patient’s residence was assigned as the location in which the ART procedure was performed.

^§^ U.S. births include nonresidents (*24*).

^¶^ To protect confidentiality, cells with values from 1–4 are suppressed, as are data that can be used to derive cell values of 1–4. These values are included in totals.

** In three states, >2% of residency information was missing for procedures performed: California (2.7%), Georgia (2.1%), and Hawaii (2.1%). Overall, residency information was missing for 963 (0.6%) procedures performed and 366 (0.7%) live-birth deliveries.

Nationally, ART contributed to approximately 4.7% (range: 1.2% in Puerto Rico to 13.4% in Massachusetts) and 5.0% (range: 1.1% in Puerto Rico to 14.7% in Massachusetts) of all preterm and very preterm infants, respectively (Table 6). In three reporting areas (Connecticut, Massachusetts, and New Jersey), >10% of all preterm and very preterm infants were conceived with ART. As with low birthweight, the percentage of preterm and very preterm infants was higher among ART-conceived infants than among the total birth population. Among ART-conceived infants, 33.2% were born preterm (range: 18.9% in the District of Columbia to 45.9% in Puerto Rico), compared with 11.3% among all infants (range: 8.5% in California to 16.0% in Mississippi). Approximately 5.9% of ART-conceived infants were very preterm (range: 2.2% in the District of Columbia to 12.0% in North Dakota), compared with 1.9% among all infants (range: 1.3% in California, Idaho, and Utah to 2.9% in Alabama).

**TABLE 6:** Number, percentage, and proportion of infants born with use of assisted reproductive technology,* by preterm gestational age category and female patient’s reporting area of residence^†^ at time of treatment — United States and Puerto Rico, 2014

Patient’s reporting area of residence	<37 weeks (PTB)			<32 weeks (VPTB)		
	ART infants	All infants^§^	Proportion of ART PTB infants among all PTB infants (%)	ART infants	All infants^§^	Proportion of ART VPTB infants among all VPTB infants (%)
	No. (%)	No. (%)		No. (%)	No. (%)	
Alabama	190 (41.2)	9,051 (15.2)	2.1	35 (7.6)	1,719 (2.9)	2.0
Alaska	20 (35.7)	1,148 (10.1)	1.7	6 (10.7)	158 (1.4)	3.8
Arizona	367 (35.7)	10,100 (11.6)	3.6	59 (5.7)	1,595 (1.8)	3.7
Arkansas	73 (37.2)	4,857 (12.6)	1.5	12 (6.1)	743 (1.9)	1.6
California^¶^	2,490 (29.4)	42,672 (8.5)	5.8	458 (5.4)	6,621 (1.3)	6.9
Colorado	353 (36.0)	6,627 (10.1)	5.3	50 (5.1)	1,022 (1.6)	4.9
Connecticut	390 (29.7)	3,410 (9.4)	11.4	70 (5.3)	600 (1.7)	11.7
Delaware	43 (19.1)	1,389 (12.7)	3.1	9 (4.0)	310 (2.8)	2.9
District of Columbia	68 (18.9)	1,269 (13.3)	5.4	8 (2.2)	247 (2.6)	3.2
Florida	1,018 (35.3)	28,724 (13.1)	3.5	204 (7.1)	5,024 (2.3)	4.1
Georgia^¶^	557 (37.8)	16,504 (12.6)	3.4	98 (6.6)	3,003 (2.3)	3.3
Hawaii^¶^	175 (39.2)	2,236 (12.1)	7.8	40 (9.0)	350 (1.9)	11.4
Idaho	83 (38.1)	2,239 (9.8)	3.7	8 (3.7)	298 (1.3)	2.7
Illinois	1361 (33.5)	18,760 (11.8)	7.3	248 (6.1)	3,438 (2.2)	7.2
Indiana	305 (37.5)	9,233 (11.0)	3.3	43 (5.3)	1,492 (1.8)	2.9
Iowa	208 (33.1)	4,471 (11.3)	4.7	38 (6.0)	779 (2.0)	4.9
Kansas	136 (37.8)	4,279 (10.9)	3.2	25 (6.9)	635 (1.6)	3.9
Kentucky	216 (42.7)	7,079 (12.6)	3.1	46 (9.1)	1,169 (2.1)	3.9
Louisiana	244 (45.1)	9,793 (15.2)	2.5	57 (10.5)	1,730 (2.7)	3.3
Maine	45 (30.8)	1,151 (9.1)	3.9	8 (5.5)	173 (1.4)	4.6
Maryland	520 (26.6)	9,051 (12.2)	5.7	102 (5.2)	1,620 (2.2)	6.3
Massachusetts	945 (27.8)	7,033 (9.8)	13.4	163 (4.8)	1,112 (1.5)	14.7
Michigan	587 (39.2)	13,547 (11.8)	4.3	103 (6.9)	2,401 (2.1)	4.3
Minnesota	372 (31.8)	7,000 (10.0)	5.3	60 (5.1)	1,161 (1.7)	5.2
Mississippi	108 (43.7)	6,193 (16.0)	1.7	19 (7.7)	1,085 (2.8)	1.8
Missouri	305 (35.1)	8,836 (11.7)	3.5	48 (5.5)	1,409 (1.9)	3.4
Montana	50 (39.1)	1,426 (11.5)	3.5	13 (10.2)	227 (1.8)	5.7
Nebraska	153 (43.7)	2,852 (10.6)	5.4	29 (8.3)	482 (1.8)	6.0
Nevada	214 (38.7)	4,678 (13.0)	4.6	32 (5.8)	730 (2.0)	4.4
New Hampshire	87 (29.5)	1,116 (9.1)	7.8	19 (6.4)	180 (1.5)	10.6
New Jersey	1,208 (32.1)	11,718 (11.3)	10.3	204 (5.4)	2,000 (1.9)	10.2
New Mexico	45 (34.9)	2,975 (11.4)	1.5	11 (8.5)	410 (1.6)	2.7
New York	1,918 (29.7)	25,398 (10.6)	7.6	335 (5.2)	4,023 (1.7)	8.3
North Carolina	516 (33.3)	14,550 (12.0)	3.5	81 (5.2)	2,929 (2.4)	2.8
North Dakota	55 (38.7)	1,126 (9.9)	4.9	17 (12.0)	182 (1.6)	9.3
Ohio	601 (36.1)	16,755 (12.0)	3.6	102 (6.1)	3,116 (2.2)	3.3
Oklahoma	172 (41.1)	6,881 (12.9)	2.5	33 (7.9)	1,098 (2.1)	3.0
Oregon	239 (35.7)	4,228 (9.3)	5.7	37 (5.5)	624 (1.4)	5.9
Pennsylvania	661 (27.1)	15,015 (10.6)	4.4	121 (5.0)	2,684 (1.9)	4.5
Puerto Rico	61 (45.9)	5,223 (15.2)	1.2	9 (6.8)	819 (2.4)	—**
Rhode Island	56 (24.1)	1,107 (10.2)	5.1	9 (3.9)	193 (1.8)	4.7
South Carolina	222 (40.7)	7,765 (13.5)	2.9	39 (7.1)	1,432 (2.5)	2.7
South Dakota	46 (36.5)	1,346 (11.0)	3.4	7 (5.6)	192 (1.6)	3.6
Tennessee	254 (40.1)	10,221 (12.5)	2.5	43 (6.8)	1,593 (2.0)	2.7
Texas	2,206 (42.1)	49,527 (12.4)	4.5	431 (8.2)	7,738 (1.9)	5.6
Utah	280 (38.8)	5,196 (10.2)	5.4	42 (5.8)	668 (1.3)	6.3
Vermont	40 (37.4)	527 (8.6)	7.6	7 (6.5)	95 (1.5)	7.4
Virginia	598 (27.8)	11,098 (10.7)	5.4	101 (4.7)	2,017 (2.0)	5.0
Washington	360 (27.8)	8,505 (9.6)	4.2	42 (3.2)	1,297 (1.5)	3.2
West Virginia	38 (37.6)	2,683 (13.2)	1.4	— (—)**	449 (2.2)	—**
Wisconsin	273 (34.7)	6,854 (10.2)	4.0	57 (7.2)	1,119 (1.7)	5.1
Wyoming	14 (25.9)	885 (11.5)	1.6	— (—)**	141 (1.8)	—**
**Total**	**21,546 (33.2)**	**456,307 (11.3)**	**4.7**	**3,846 (5.9)**	**76,332 (1.9)**	**5.0**

**Abbreviations:** ART = assisted reproductive technology; PTB = preterm birth; VPTB = very preterm birth.

* ART totals include infants conceived from ART procedures performed in 2013 and born in 2014 and infants conceived from ART procedures performed in 2014 and born in 2014. Total ART births exclude nonresidents and include only infants with gestational age data available.

^†^ In cases of missing residency data (0.6%), the patient’s residence was assigned as the location in which the ART procedure was performed.

^§^ U.S. births include nonresidents (*24*).

^¶^ In three states, >2% of residency information was missing for procedures performed: California (2.7%), Georgia (2.1%), and Hawaii (2.1%). Overall, residency information was missing for 963 (0.6%) procedures performed and 366 (0.7%) live-birth deliveries.

** To protect confidentiality, cells with values from 1–4 are suppressed, as are data that can be used to derive cell values of 1–4. These values are included in totals.

The percentage of ART-conceived infants who were low birthweight was 8.9% (range: 3.2% in some states to 16.1% in Vermont) among singletons, 55.2% (range: 38.5% in Delaware to 77.8% in Alaska) among twins, and 95.3% (range: 0% in some states to 100% in several reporting areas) among triplets and higher-order infants; the corresponding percentage among all infants born was 6.3% (range: 4.6% in Alaska, North Dakota, and Oregon to 9.5% in Puerto Rico) among singletons, 55.2% (range: 46.1% in Alaska to 65.6% in Mississippi) among twins, and 94.5% (range: 88.4% in Alabama to 100% in several reporting areas) among triplets and higher-order infants (Table 7).

**TABLE 7:** Percentages of low birthweight infants (<2,500 g) among infants born with assisted reproductive technology* and all U.S. infants, by plurality and female patient’s reporting area of residence^†^ at time of treatment — United States and Puerto Rico, 2014

Patient’s reporting area of residence	ART singleton infants (%)	All singleton infants^§^ (%)	ART twin infants^¶^ (%)	All twin infants^§^ (%)	ART triplets and higher-order infants^¶^ (%)	All triplets and higher-order infants^§^ (%)
Alabama	9.6	8.1	59.6	62.2	—**	88.4
Alaska	—^††^	4.6	77.8	46.1	—^§§^	—**
Arizona	9.9	5.5	53.2	53.5	100.0	91.2
Arkansas	10.2	7.3	59.7	59.3	—**	100.0
California^¶¶^	8.0	5.1	52.9	51.4	94.4	94.1
Colorado	11.2	7.1	64.8	59.7	100.0	96.8
Connecticut	8.2	5.6	51.2	52.0	—**	100.0
Delaware	7.0	7.0	38.5	49.8	—^§§^	—**
District of Columbia	8.5	7.9	40.3	57.0	—**	—**
Florida	10.4	6.9	52.1	57.2	93.7	95.4
Georgia^¶¶^	9.9	7.6	58.6	60.0	90.0	94.7
Hawaii^¶¶^	10.9	6.2	65.6	59.9	—**	100.0
Idaho	7.8	5.0	62.6	53.3	—**^,††^	—**
Illinois	9.1	6.1	54.4	55.5	98.4	100.0
Indiana	6.0	6.2	54.0	55.2	91.7	100.0
Iowa	6.8	5.0	55.5	51.0	—**^,††^	100.0
Kansas	6.6	5.5	62.5	52.5	—**	100.0
Kentucky	9.1	7.1	56.5	56.6	100.0	100.0
Louisiana	9.5	8.4	63.4	64.9	—**	100.0
Maine	—^††^	5.6	58.3	57.3	—**^,††^	—^††^
Maryland	10.9	6.7	58.4	55.1	—**	93.9
Massachusetts	8.5	5.3	50.6	54.0	95.2	94.0
Michigan	9.9	6.5	54.1	54.5	100.0	100.0
Minnesota	8.1	4.9	51.0	48.8	—**	100.0
Mississippi	10.5	9.3	50.5	65.6	—**	100.0
Missouri	8.7	6.3	58.3	57.8	—**	100.0
Montana	10.8	5.8	61.3	51.7	—^§§^	—**
Nebraska	6.0	5.0	50.6	50.3	—**	100.0
Nevada	10.3	6.6	54.4	58.8	—**^,††^	—^††^
New Hampshire	10.4	5.2	55.6	52.3	—**^,††^	—**
New Jersey	8.5	5.8	57.2	55.7	100.0	92.4
New Mexico	11.3	7.4	59.5	62.9	—**	—^††^
New York	9.0	5.9	58.0	54.6	88.7	93.3
North Carolina	9.0	7.1	50.9	54.2	—**	94.8
North Dakota	7.8	4.6	54.8	50.0	—**^,††^	—^††^
Ohio	8.9	6.6	55.0	56.0	98.0	95.3
Oklahoma	8.9	6.4	56.6	54.9	—**	100.0
Oregon	8.3	4.6	48.3	48.7	—**	100.0
Pennsylvania	8.0	6.5	54.9	52.4	100.0	92.8
Puerto Rico	15.3	9.5	64.7	65.3	—**	95.2
Rhode Island	6.9	5.5	41.5	49.4	—**^,††^	—**^,††^
South Carolina	10.1	7.6	57.8	59.0	—**	100.0
South Dakota	—^††^	5.0	44.2	50.1	—**	—**
Tennessee	9.7	7.2	59.3	60.1	—**	100.0
Texas	10.2	6.5	59.7	57.5	93.5	98.0
Utah	9.2	5.2	58.5	51.3	—**	100.0
Vermont	16.1	5.3	50.0	46.4	—**^,††^	—**
Virginia	8.9	6.1	51.1	52.0	100.0	100.0
Washington	7.9	5.0	46.8	51.3	90.5	88.5
West Virginia	7.8	7.2	53.1	62.9	—**^,††^	—^††^
Wisconsin	5.2	5.7	53.7	53.0	—**	100.0
Wyoming	—^††^	7.3	—**	61.0	—**^,††^	—**
**Total**	**8.9**	**6.3**	**55.2**	**55.2**	**95.3**	**94.5**

**Abbreviation:** ART = assisted reproductive technology.

* ART totals include infants conceived from ART procedures performed in 2013 and born in 2014 and infants conceived from ART procedures performed in 2014 and born in 2014. Total ART births exclude nonresidents and only include infants with birthweight data available.

^†^ In cases of missing residency data (0.6%), the patient’s residence was assigned as the location in which the ART procedure was performed.

^§^ U.S. births include nonresidents (*24*).

^¶^ Includes only the number of infants live-born in a multiple-birth delivery. For example, if three infants were born in a live-birth delivery and one of the three infants was stillborn, the total number of live-born infants would be two. However, the two infants still would be counted as triplets.

** Estimates on the basis of N <20 in the denominator have been suppressed because such rates are considered unstable.

^††^ To protect confidentiality, cells with values from 1–4 are suppressed, as are data that can be used to derive cell values of 1–4. These values are included in totals. For all births, cell values are suppressed for cell/numerator <10 or if a total/subtotal for a range of cells with a single suppressed member.

^§§^ Estimates not calculated because N = 0 for the denominator.

^¶¶^ In three states, >2% of residency information was missing for procedures performed: California (2.7%), Georgia (2.1%), and Hawaii (2.1%). Overall, residency information was missing for 963 (0.6%) procedures performed and 366 (0.7%) live-birth deliveries.

The percentage of ART-conceived infants who were born preterm was 13.2% among singletons (range: 7.5% in Rhode Island to 23.4% in West Virginia), 62.2% among twins (range: 33.3% in some states to 81.4% in Mississippi), and 98.2% among triplets and higher-order infants (range: 50.0% in some states to 100% in several reporting areas); the corresponding percentage among all infants was 9.7% for singletons (range: 1.7% in the District of Columbia to 14.2% in Mississippi), 56.6% for twins (range: 47.2% in Vermont to 66.9% in Wyoming), and 93.8% for triplets and higher-order infants (range: 52.2% in Hawaii to 100% in several reporting areas) (Table 8).

**TABLE 8:** Percentages of preterm (<37 weeks) infants among infants born with the use of assisted reproductive technology* and all U.S. infants, by plurality and female patient’s reporting area of residence^†^ at time of treatment — United States and Puerto Rico, 2014

Patient’s reporting area of residence	ART singleton infants (%)	All singleton infants^§^ (%)	ART twin infants^¶^ (%)	All twin infants^§^ (%)	ART triplets and higher-order infants^¶^ (%)	All triplets and higher order-infants^§^ (%)
Alabama	14.5	13.5	66.5	62.4	—**	100.0
Alaska	—^††^	8.6	80.0	55.9	—^§§^	—**
Arizona	14.8	10.1	64.8	58.6	100.0	94.1
Arkansas	13.6	11.2	75.0	57.4	—**^,††^	76.7
California^¶¶^	12.1	7.0	56.9	52.3	98.2	94.9
Colorado	15.6	8.5	63.0	57.4	100.0	88.9
Connecticut	11.9	7.5	58.1	50.6	—**	71.1
Delaware	13.6	11.4	61.5	54.0	—^§§^	—**
District Of Columbia	9.9	1.7	—^††^	52.9	—**	—**
Florida	14.9	11.5	59.6	56.6	100.0	93.1
Georgia^¶¶^	14.9	10.9	70.2	58.0	100.0	90.9
Hawaii^¶¶^	13.1	10.6	64.4	54.8	—**	52.2
Idaho	12.9	8.2	65.7	60.9	—**^,††^	—**
Illinois	13.9	9.8	62.0	59.4	100.0	96.8
Indiana	11.2	9.3	64.9	56.9	100.0	100.0
Iowa	11.0	9.4	70.9	59.3	—**^,††^	100.0
Kansas	11.6	9.3	73.8	58.3	—**	87.0
Kentucky	11.4	11.0	68.8	58.7	100.0	100.0
Louisiana	17.7	13.2	74.0	66.7	—**	93.6
Maine	12.6	7.3	62.5	53.4	—**^,††^	93.6
Maryland	13.5	10.4	60.5	57.7	—**	100.0
Massachusetts	11.9	7.6	59.3	58.3	93.3	96.4
Michigan	16.7	10.1	65.6	57.2	100.0	89.9
Minnesota	11.6	8.3	59.3	52.3	—**	100.0
Mississippi	16.0	14.2	81.4	65.5	—**	66.7
Missouri	13.3	9.9	62.0	62.1	—**	98.5
Montana	10.8	9.9	68.3	55.3	—^§§^	—**
Nebraska	9.6	8.9	72.6	58.2	—**	87.7
Nevada	15.4	11.5	73.4	60.3	—**^,††^	92.3
New Hampshire	10.9	7.4	59.3	52.0	—**^,††^	—**
New Jersey	12.6	9.3	63.9	54.8	100.0	95.5
New Mexico	14.8	10.2	71.4	59.7	—**^,††^	100.0
New York	12.7	8.9	58.6	53.3	92.4	92.0
North Carolina	14.2	10.5	58.2	52.9	—**	89.7
North Dakota	10.4	8.3	71.0	55.0	—**^,††^	68.2
Ohio	13.0	10.2	59.3	57.2	100.0	89.8
Oklahoma	13.1	11.3	67.2	61.4	—**	93.4
Oregon	16.3	7.6	54.3	52.6	—**	93.0
Pennsylvania	10.5	8.8	53.9	55.1	100.0	74.8
Puerto Rico	18.6	14.1	64.7	62.3	—**	100.0
Rhode Island	7.5	8.7	—^††^	52.0	—**^,††^	—**
South Carolina	17.3	11.8	70.3	57.7	—**^,††^	100.0
South Dakota	11.8	9.4	61.5	55.5	—**	—**
Tennessee	15.5	10.8	71.3	61.1	—**	93.1
Texas	17.2	10.7	70.7	59.9	100.0	94.5
Utah	12.4	8.3	61.9	56.3	—**	100.0
Vermont	12.5	6.9	62.5	47.2	—**^,††^	—**
Virginia	11.0	9.1	61.2	52.6	100.0	91.3
Washington	10.5	8.2	54.3	52.3	100.0	80.8
West Virginia	23.4	11.4	58.8	64.2	—**^,††^	57.1
Wisconsin	11.4	8.6	67.0	54.6	—**	96.7
Wyoming	15.2	9.6	—^††^	66.9	—**^,††^	—**
**Total**	**13.2**	**9.7**	**62.2**	**56.6**	**98.2**	**93.8**

**Abbreviation:** ART = assisted reproductive technology.

* ART totals include infants conceived from ART procedures performed in 2013 and born in 2014 and infants conceived from ART procedures performed in 2014 and born in 2014. Total ART births exclude nonresidents and includes only infants with gestational age data available.

^†^ In cases of missing residency data (0.6%), the patient's residence was assigned as the location in which the ART procedure was performed.

^§^ U.S. births include nonresidents (*24*).

^¶^ Includes only the number of infants live-born in a multiple-birth delivery. For example, if three infants were born in a live-birth delivery and one of the three infants was stillborn, the total number of live-born infants would be two. However, the two infants still would be counted as triplets.

** Estimates on the basis of N <20 in the denominator have been suppressed because such rates are considered unstable.

^††^ To protect confidentiality, cells with values from 1–4 are suppressed, as are data that can be used to derive cell values of 1–4. These values are included in totals.

^§§^ Estimates not calculated because N = 0 for the denominator.

^¶¶^ In three states, >2% of residency information was missing for procedures performed: California (2.7%), Georgia (2.1%), and Hawaii (2.1%). Overall, residency information was missing for 963 (0.6%) procedures performed and 366 (0.7%) live-birth deliveries.

## Discussion

### Overview

The use of ART has increased substantially in the United States since the beginning of ART surveillance. In 1996 (the first full year for which ART data were reported to CDC), a total of 20,597 infants were born from 64,036 ART procedures (*25*). Since then, the number of procedures reported to CDC and the number of infants born from ART procedures have approximately tripled. Several changes can be observed in ART use and outcomes since the preceding reporting year in 2013 (*26*). The rate of ART use as measured by procedures performed per 1 million women of reproductive age (15–44 years) increased from 2,521 to 2,647. Among women aged <35 years, the average number of embryos transferred decreased (from 1.8 to 1.7) and correspondingly, the percentage of eSET increased (from 21.4% to 28.5%). Overall, the percentage of ART-conceived infants born in multiple-birth deliveries decreased from 41.1% to 39.4%, the percentage of ART-conceived twin infants decreased from 39.2% to 37.5%, and the percentage of ART-conceived triplets and higher-order infants decreased from 1.9% to 1.8%. However, the contribution of ART-conceived twin infants to all twin infants and the contribution of ART-conceived preterm infants to all preterm infants remained unchanged at approximately 18.0% and 4.7%, respectively.

The contribution of ART on rates of multiple births and poor birth outcomes remained substantial in 2014, as approximately 39.0% of ART-conceived infants were born in multiple births (compared with only 3.5% of infants among the total birth population). The contribution of ART-conceived infants to all triplets and higher-order infants increased slightly from 25.2% in 2013 to 26.4% in 2014. ART-conceived twins accounted for approximately 95.3% (24,514 of 25,714) of all ART-conceived infants born in multiple-birth deliveries. On average, approximately two embryos were transferred among women aged <35 years, even though single embryo transfers have been associated with better perinatal outcomes among the majority of women in this age group (*27*,*28*). Although the rate of eSET procedures was still relatively low among women aged <35 years, from 2013 to 2014, the eSET rate increased from 21.4% to 28.5%. This is the largest annual percentage increase (33.6%) in eSET rate in the U.S ever detected through NASS. The percentage of low birthweight and preterm birth was substantially higher among ART-conceived infants (27.8% and 33.2%, respectively) than among all infants (8.0% and 11.3%, respectively). Among ART-conceived infants, twins and triplets and higher-order infants were more likely than singletons to be born preterm (more than 4.5 times and seven times, respectively). Although infants conceived with ART accounted for approximately 1.6% of total births in the United States in 2014, the proportions of twins and triplets and higher-order infants attributable to ART were 18.0% and 26.4%, respectively.

Comparable data on ART use and embryo transfer practices from 17 European countries indicate that in 2011, ART use as defined by the number of procedures performed per 1 million women of reproductive age was 6,556; this was approximately 2.7 times higher than the rate in the United States in 2011 (*29*,*30*). Percentages of single-embryo transfers (eSET rates are not reported) varied widely in Europe, and a few countries reported a single-embryo transfer rate of over 50.0%. Overall, in these 17 reporting countries, approximately 81.0% of all IVF deliveries were singleton deliveries, compared with 72.0% in the United States (*29*,*30*).

### Variations by Reporting Area

ART use (as measured by the number of ART procedures performed per 1 million women of reproductive age) varied widely by reporting area: residents of Connecticut, Delaware, Hawaii, Illinois, Maryland, Massachusetts, New Hampshire, New Jersey, New York, Pennsylvania, Rhode Island, Virginia, and the District of Columbia had higher ART use than the national level. Although some women who used ART might have been aged >44 years, this measure is still useful as a proxy indicator of ART use in each reporting area. Importantly, residents of California, Illinois, Massachusetts, New Jersey, New York, and Texas accounted for nearly half (47.0%) of all ART-conceived infants. The large number of ART procedures performed in these states is a result of the large size of the general population (California, Texas), higher ART use (Massachusetts, New Jersey), or both (New York, Illinois). The contribution of ART to all infants born was 4.7% in Massachusetts and 1.7% in California.

Such differences might be explained in part by variations in state health insurance coverage. A total of 15 states (Arkansas, California, Connecticut, Hawaii, Illinois, Louisiana, Maryland, Massachusetts, Montana, New Jersey, New York, Ohio, Rhode Island, Texas, and West Virginia) have passed legislation mandating insurance coverage for fertility treatments, although not all mandates require coverage for ART; mandates from four of these states (Illinois, Massachusetts, New Jersey, and Rhode Island) include comprehensive coverage for at least four cycles of IVF.^†^ Three of the four states with comprehensive mandates (Illinois, Massachusetts, and New Jersey) had rates of ART use that were at least 50.0% higher than the national level. This type of mandated insurance has been associated with greater use of ART (*31*–*33*). Linkage of NASS data with birth certificate data in three states indicated that Massachusetts had a higher overall rate of ART use compared with Florida and Michigan, which do not have a comprehensive coverage mandate for ART (*33*).

### Elective Single-Embryo Transfer Rates

According to the American Society of Reproductive Medicine (ASRM) and SART, eSET is recommended for favorable prognosis patients (patients who underwent their first IVF cycle and had extra embryos cryopreserved, patients with previous successful IVF procedures, or patients who are recipients of oocytes from a donor aged <35 years) (*34*). The guidelines issued by ASRM/SART on the number of embryos transferred have been revised several times (*35*–*39*). However, ASRM/SART guidelines on the number of embryos transferred allow for up to two embryos to be transferred even among favorable prognosis patients with patient counseling regarding risks for multifetal pregnancies (*35*). A high number of double-embryo transfers occur among patients who might otherwise appear to be good candidates for transferring one embryo (*40*). Reducing the number of embryos transferred from two to one among those patients who have a good chance of pregnancy and live birth with single-embryo transfers will lower ART-conceived twin rates (*40*,*41*).

Among women aged <35 years, the percentage of eSET procedures was higher (28.5% nationally) than among older age groups (16.7% among women aged 35–37 years and 4.7% among women aged >37 years nationally) and varied widely among reporting areas (range: 4.3% to 67.9%). The national percentage of eSET increased from 7.4% in 2009 to 28.5% in 2014 among women aged <35 years (*26*). From 2013 to 2014, the national percentage of eSET among women aged <35 years increased by approximately 33.0%. The percentage of eSET is still lower in the United States than in countries that impose restrictions on the number of embryos transferred and provide extensive public funding for ART services (*42*). The eSET rates are influenced by many factors (e.g., patient’s age, diagnostic factors, and treatment costs); ART treatment costs are high and typically paid out-of-pocket by the patient. In the United States, even where mandated, coverage for infertility treatment can vary in scope (*31*). In the four states with mandatory comprehensive insurance coverage for ART, the eSET percentage among women aged <35 years was higher than the national average (28.5%) in Massachusetts (51.5%), New Jersey (34.7%), and Rhode Island (34.5%) but lower in Illinois (27.2%). Because ART procedures are expensive (out-of-pocket costs to achieve a live birth estimated at $27,000 for patients with no insurance), broad insurance mandates for IVF might increase the use of eSET and decrease multiple-embryo transfer procedures (*32*,*43*,*44*). Wider acceptance and use of eSET procedures still face considerable barriers in the United States and might require strengthening the guidelines on embryo transfer practices along with expansion of insurance coverage for ART services (*40*,*41*,*44*,*45*).

### ART Multiple-Birth Infants

Singleton live-birth deliveries have lower risks than multiple-birth deliveries for adverse birth outcomes such as prematurity, low birthweight, disability, and death (*46*–*48*). To optimize healthy birth outcomes, the transfer of fewer embryos should be encouraged, where appropriate, taking into consideration the patient’s age and prognosis (*27*). The percentage of ART-conceived multiple-birth infants in the United States decreased from 53.1% in 2000 to 39.4% in 2014 (*49*). A substantial decrease was noted in the percentage of ART-conceived triplets and higher-order infants (from 8.9% in 2000 to 1.8% in 2014), and a smaller decrease was noted in the percentage of ART-conceived twins (from 44.2% in 2000 to 37.5% in 2014).

In the past, the slow decrease in twin-infant birth rates among women who undergo ART procedures was largely attributable to gradual increases in eSET rates (*40*,*41*). From 2013 to 2014, an historically large increase of 33.0% in national eSET rate was observed. Despite this increase in eSET use, ART-conceived twin infants still accounted for approximately 40.0% of all ART-conceived infants in 2014, and on average, 1.7 embryos were transferred among favorable prognosis patients aged <35 years. In addition to embryo transfer practices, high ART-conceived twin rates also might be partially explained by a desire for more than one child among couples experiencing infertility who might believe that the benefits of a multiple-gestation pregnancy outweigh the risks (*50*–*52*). Therefore, understanding the perspective of couples undergoing infertility treatments regarding multiple-gestation pregnancies and multiple births is an important consideration. Although a major barrier to the greater use of eSET might be the high out-of-pocket cost of ART, the use and acceptance of eSET among younger patients with good prognosis can be promoted through patient education. The findings in this report indicate that ART-conceived twins and higher-order infants were more than 4.5 times and seven times more likely, respectively, to be born preterm than were ART-conceived singletons. Studies indicate that patient education focusing on maternal and perinatal morbidity and mortality, as well as the economic costs of twin gestation, has been effective in reducing the preference for twins among patients (*53*–*55*).

The economic costs of multiple births also underscore the importance of efforts to reduce ART-related multiple births. In 2013, the mean health care cost to patients and insurers was estimated to be $26,922 for ART-conceived singleton deliveries, $115,238 for ART-conceived twins, and $434,668 for ART-conceived triplets and higher-order infants (*56*). Transferring two embryos is associated with a slight increase in birth rate but a greater increase in the twin birth rate as compared with transferring a single embryo (*27*,*57*). However, transferring two embryos sequentially (single-embryo transfer over two sequential procedures) has similar cumulative live-birth rates and lower twin delivery rates than transferring two embryos in a single procedure and might be a cost-effective transfer approach (*58*–*60*). As a result of the data on the higher economic costs of maternal and neonatal complications that occur with multiple births, insurance companies could consider expanded coverage for ART services that also includes clinically appropriate use of eSET and other limitations placed on the number of embryos transferred (*41*,*45*,*56*). Evidence from other countries suggests that greater insurance coverage for ART when combined with restrictions on the number of embryos transferred per cycle can reduce multiple births (*45*).

### ART Low Birthweight Infants and Preterm Births

The percentage of low birthweight and very low birthweight infants was higher among ART-conceived infants than among infants in the total birth population. Three states (Connecticut, Massachusetts, and New Jersey) that had large numbers of ART procedures and births also had high ART contribution (>10%) to both categories of low birthweight and preterm births. In the United States, the contribution of ART to preterm births, the majority of which are also low birthweight, is a key concern. Fertility treatments, both ART and controlled ovarian stimulations, contribute substantially to preterm births (*47*,*61*). Preterm births are a leading cause of infant mortality and morbidity; preterm infants are at increased risk for death and have more health and developmental problems than full-term infants (*47*,*62*–*64*). The health risks associated with preterm birth have contributed to increased health care costs. The societal economic costs associated with all preterm births in the United States was last reported in 2005 and was estimated at $26 billion annually ($51,600 per infant born preterm) (*47*). In 2012, the societal economic costs associated with ART-conceived preterm infants in the United States was estimated at approximately $1.3 billion (*65*).

In addition to the known multiple-birth risks associated with ART, even singleton infants conceived from ART procedures are at increased risk for low birthweight and preterm delivery. In 2014, of all ART-conceived singleton infants, 9.0% were low birthweight, compared with 6.3% of infants in the total birth population. The percentage of ART-conceived singletons born preterm was 13.2%, compared with 9.7% of infants in the total U.S. birth population. Therefore, adverse infant health outcomes (e.g., low birthweight and preterm delivery) among singletons also should be considered when assessing the effects of ART.

## Limitations

The findings in this report are subject to at least five limitations. First, ART surveillance data were reported for each ART procedure performed rather than for each patient who used ART. Second, because patients can achieve a successful pregnancy after undergoing multiple procedures, the procedure-specific success rates reported here underestimate the true per-patient success rates. Third, prematurity and low birthweight could be associated with factors contributing to underlying infertility and not entirely to ART procedures. Fourth, approximately 8.0% of fertility clinics that performed ART in 2014 did not report their data to CDC, and these clinics might have had results differing from reporting clinics. Finally, NASS lacks data on embryo quality, which influences the use of eSET among favorable prognosis patients aged <35 years; however, having extra embryos available for cryopreservation has been shown to be a good predictor of embryo quality.

## Conclusion

Since 1996, the number of ART procedures performed in the United States and the number of infants born as a result of these procedures nearly tripled. With this increasing use, ART-conceived infants represented 1.6% of infants born in the United States in 2014 and noticeably contributed to the prevalence of low birthweight and preterm deliveries, as approximately two fifths of ART-conceived infants were multiple-birth deliveries. Furthermore, among ART-conceived infants, although the percentage of triplets or higher-order infants has decreased since 2000, the percentage of twin infants has remained persistently high. Therefore, the impact of ART on poor birth outcomes remains substantial. This report documents the rates and contribution of ART to multiple-birth deliveries, low birthweight, and preterm birth by patient’s reporting area of residence. It also highlights the differences in rates of low birthweight and prematurity between ART-conceived infants and all infants in the total birth population. These findings allow state health departments to monitor the extent of ART-related adverse perinatal outcomes among singletons, twins, and triplets and higher-order infants in their reporting areas.

Comprehensive insurance coverage of ART can help increase access to fertility treatments (*45*). Increased use of ART in reporting areas with insurance mandates also can result in higher numbers of ART-conceived multiple-birth deliveries. The findings in this report indicate that ART use was higher than the national rate in all four states with mandated comprehensive insurance coverage. Three of these four states had use rates exceeding 1.5 times the national rate and among these three states, two had a percentage of multiple births that was lower than the national average. Further, in three of these states, among patients aged <35 years, the average number of embryos transferred was less than the national rate and the rate of eSET was higher than the national rate. Further research is needed to ascertain the influence of state health insurance mandates on ART use, embryo transfer practices, infant outcomes, and economic and out-of-pocket patient costs of multiple births (*28*,*34*,*40*,*41*). Addressing the risk for multiple-birth deliveries also requires understanding the perspectives of couples undergoing infertility treatments who might view a multiple birth, especially twins, as an acceptable or desired outcome or might lack awareness of the increased risks associated with multiple births to mothers and infants. Although the majority of clinicians acknowledge that the birth of a healthy singleton is the best outcome of ART, they might be sensitive to patient perspectives (*34*,*35*). Clinicians need to be aware of ongoing efforts to limit the number of embryos transferred to reduce the rate of multiple births, particularly twins, and encourage wider implementation of eSET, when clinically appropriate, as mechanisms of promoting singleton infant births among ART-conceived pregnancies (*27*,*34*,*41*).

CDC has outlined a public health oriented strategy for detection, prevention, and management of infertility, including improving ART practice and outcomes, through coordinated efforts of government and nongovernment organizations. The effort involves federal, state, and local agencies; the scientific community; health care professionals; insurance providers; employers; industry; nonprofit organizations; and organizations representing people coping with infertility (*66*). Of public health importance is the role of infertility treatment on adverse birth outcomes, primarily because of higher rates of multiple births. ART only partially explains the overall prevalence of these adverse outcomes in the United States. Other factors influencing multiple births include maternal age at conception and non-ART fertility treatments (*47*,*61*,*67*). The older age of women giving birth accounted for a substantial increase in twins during 1980–2009 (*67*). The risk for multiple gestations associated with non-ART fertility treatments (i.e., controlled ovarian stimulation such as superovulation-intrauterine insemination and conventional ovulation induction) is less well-documented than those from ART procedures, as clinics are only mandated to report data on ART use. Further efforts also are needed to monitor the use of non-ART fertility treatments and their role in the increasing number of multiple-birth deliveries (*47*,*61*). In 2011, approximately 19.0% of twin births in the United States were attributable to non-IVF fertility treatments (*61*). Multiple gestations resulting from non-ART fertility treatments also might contribute to preterm births (*47*,*61*). Additional research is needed to identify the causes and consequences of preterm births that occur because of infertility treatments and to develop and institute guidelines to reduce the number of multiple gestations (*47*,*61*). However, studies have demonstrated that singleton infants conceived with ovulation stimulation are more likely than naturally conceived infants to be small for gestational age (*68*). CDC is monitoring the prevalence of non-ART fertility treatment use and resultant outcomes among women who had live births in several states participating in the Pregnancy Risk Assessment Monitoring System (*69*). U.S. birth certificate data published annually by CDC’s National Center for Health Statistics plans to include information on the use of ART and non-ART treatments and birth outcomes from data year 2016. CDC also is working to improve state-based surveillance of ART, infertility, and other birth-related issues by linking data from NASS to data collected by states (i.e., birth certificate, infant death, hospital discharge, and birth defect registry information). This initiative, the States Monitoring Assisted Reproductive Technology (SMART) Collaborative,^§^ has been determined to be feasible and useful for monitoring long-term outcomes of ART (*70*,*71*). CDC will continue to provide updates of ART use in the United States as data become available.

## Footnotes

* Data regarding population size were compiled on the basis of July 1, 2014, estimates from the U.S. Census Bureau.

† Eight states (Arkansas, Connecticut, Hawaii, Illinois, Maryland, Massachusetts, New Jersey, and Rhode Island) have insurance mandates that cover at least one ART cycle. Seven states (California, Louisiana, Montana, New York, Ohio, Texas, and West Virginia) have insurance mandates that exclude IVF coverage. Information is available at http://www.resolve.org/family-building-options/insurance_coverage/state-coverage.html.

§ States Monitoring Assisted Reproductive Technology (SMART) is a collaboration between CDC and state health departments in Connecticut, Florida, Massachusetts, and Michigan. Information is available at https://www.cdc.gov/art/smart/index.html.
